# Engineered Stem Cell Clusters for Extracellular Vesicles‐Mediated Gene Delivery to Rejuvenate Chondrocytes and Facilitate Chondrogenesis in Osteoarthritis Therapy

**DOI:** 10.1002/advs.202500964

**Published:** 2025-04-25

**Authors:** Yuezhou Wu, Yubo Feng, Fei Hu, Xu Zheng, Yurun Ding, Xuesong Liu, Shicheng Huo, Zhuocheng Lyu

**Affiliations:** ^1^ Department of Bone and Joint Surgery Department of Orthopedics Renji Hospital School of Medicine Shanghai Jiaotong University 145 Middle Shandong Road Shanghai 200001 China; ^2^ Department of Spine Surgery Department of Orthopedics Renji Hospital School of Medicine Shanghai Jiaotong University 160 Pujian Road Shanghai 200001 China; ^3^ Department of Ultrasound Renji Hospital School of Medicine Shanghai Jiao Tong University 160 Pujian Road Shanghai 200001 China; ^4^ Department of Orthopedic Surgery Spine Center Changzheng Hospital Navy Medical University 415 Fengyang Road Shanghai 200001 China

**Keywords:** chondrocyte, engineered BMSC cluster, extracellular matrix, gene delivery, osteoarthritis

## Abstract

Gene therapy offers an ideal potential treatment strategy for osteoarthritis (OA). However, the safe and efficient delivery of therapeutic genes remains highly challenging because of the inactivation in direct delivery of miRNA, low transfection efficiency, and a short half‐life. This study introduced a gene therapy strategy using mesenchymal stem cells (MSCs) as a gene delivery platform and achieved the sustained delivery of therapeutic genes via engineered MSCs‐derived extracellular vesicles (EVs). The miRNA‐874‐3p is combined with an exosome‐targeting motif and transfected into bone marrow mesenchymal stem cells (BMSCs). The BMSCs^motif+miR874^ are then seeded onto hydrogel microspheres, creating the BMSC^motif+miR874^/MS system for OA treatment. In vitro experiments demonstrated that miRNA‐874‐3p not only alleviated inflammation and oxidative stress‐induced damage to chondrocytes by downregulating the NF‐κB signaling pathway, thereby rejuvenating chondrocytes, but also promoted chondrogenesis in the inflammatory microenvironment. Furthermore, the engineered BMSCs in the system demonstrated prolonged retention in vivo, thereby enabling the sustained delivery of the therapeutic gene, miRNA‐874‐3p, over an extended duration. In the rat OA model, BMSC^motif+miR874^/MS successfully delivered miRNA‐874‐3p to the articular cartilage and effectively alleviated cartilage degeneration. In conclusion, this EVs‐mediated therapeutic gene delivery approach enables miRNA‐based gene therapy a viable alternative to surgery for OA treatment and provides a novel option for gene therapy.

## Graphical abstract

BMSC^motif+miR874^/MS system is designed as a gene delivery platform utilizing engineered bone marrow mesenchymal stem cell (BMSC)‐derived extracellular vesicles (EVs) for intercellular communication to achieve sustained delivery of miRNA‐874‐3p. Rejuvenation of chondrocytes and promotion of chondrogenesis are achieved by inhibition of the NF‐κB pathway, leading to promising therapeutic effects in the early treatment of osteoarthritis (OA).

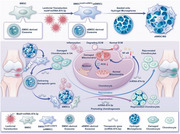

## 1 Introduction

Osteoarthritis (OA) is a chronic degenerative disease affecting over 300 million people worldwide, and its high treatment costs have become one of the major social expenditures associated with the global aging population.^[^ ^1^, ^2^ ^]^ Unfortunately, there are currently no effective interventions that can prevent or suppress the progression of OA over the long term. The hallmark of OA is the gradual loss of cartilage matrix and other pathological changes in joint components, such as osteophyte formation and synovial inflammation.^[^ ^3^, ^4^ ^]^ The pathogenesis of OA is typically associated with aging, abnormal mechanical stress, cellular metabolism, protease activity, and inflammatory changes.^[^ ^5^, ^6^ ^]^ Under the stimulation of the above factors, excessive expression of various matrix‐degrading enzymes occurs, leading to the progressive degradation of the chondrocyte extracellular matrix (ECM).^[^ ^7^ ^]^ Meanwhile, due to the continued stimulation of inflammation and oxidative stress, chondrocyte mitochondria are damaged, leading to cellular metabolic imbalance and impairing the self‐regulatory function of chondrocytes, thereby further exacerbating cellular damage and accelerating cellular senescence.^[^ ^8^ ^]^ Articular cartilage is a highly specialized tissue that lacks a vascular supply for nutrition, which limits its intrinsic repair capacity.^[^ ^9^, ^10^ ^]^ Consequently, maintaining the function of resident chondrocytes within the cartilage is crucial for joint health. Therefore, a promising strategy for treating OA would be to fundamentally address the degeneration of the chondrocyte matrix and maintain chondrocyte homeostasis.

In addition to inhibiting cellular senescence, it is also necessary to promote the differentiation of mesenchymal stem cells (MSCs) into chondrocytes, enabling the regeneration of the entire joint and further enhancing cartilage repair to counteract the progressive destruction of cartilage during the progressive stages of OA.^[^ ^11^, ^12^ ^]^ Given the large size of the human knee joint, it is unlikely that MSCs would migrate over long distances in large numbers to the injury site. Therefore, exogenous supplementation of MSCs with proliferation and differentiation potential is a commonly used solution.^[^ ^13^, ^14^, ^15^ ^]^ However, whether exogenously supplied MSCs can survive long‐term post‐transplantation and maintain functionality remains a significant challenge, particularly in severely diseased joint environments where cells may lose viability due to the lack of a favorable microenvironment or exposure to excessive mechanical stress. The hydrogel microspheres possess characteristics similar to the extracellular matrix of chondrocytes and have been widely used for the delivery of drugs, cells, or small molecules, which can provide protection for these therapeutic components and facilitate their effective delivery.^[^ ^16^ ^]^

Gene therapy, as an emerging approach, has made significant progress in recent years. For example, siRNAs and miRNAs have been shown to regulate various pathological processes during OA by influencing the expression of their target genes.^[^ ^17^, ^18^ ^]^ miRNA‐874‐3p has been reported to not only downregulate matrix metalloproteinases, thereby alleviating the degradation of the ECM, but also to be associated with chondrocyte differentiation and growth, making it a promising candidate for OA treatment.^[^ ^19^, ^20^ ^]^ However, gene therapy still faces substantial challenges in nucleic acid delivery, particularly in terms of sustained delivery and transfection efficiency.^[^ ^21^ ^]^ In recent years, a novel transfection technology that fuses miRNAs with exosome‐targeting motifs has emerged, enabling the packaging of miRNAs into extracellular vesicles (EVs) for efficient, non‐toxic delivery.^[^ ^22^, ^23^ ^]^ This sustained delivery of miRNA via EVs has the potential to overcome the challenges of limited gene delivery duration and low transfection efficiency, offering broad applications in gene therapy.

**Scheme 1:**
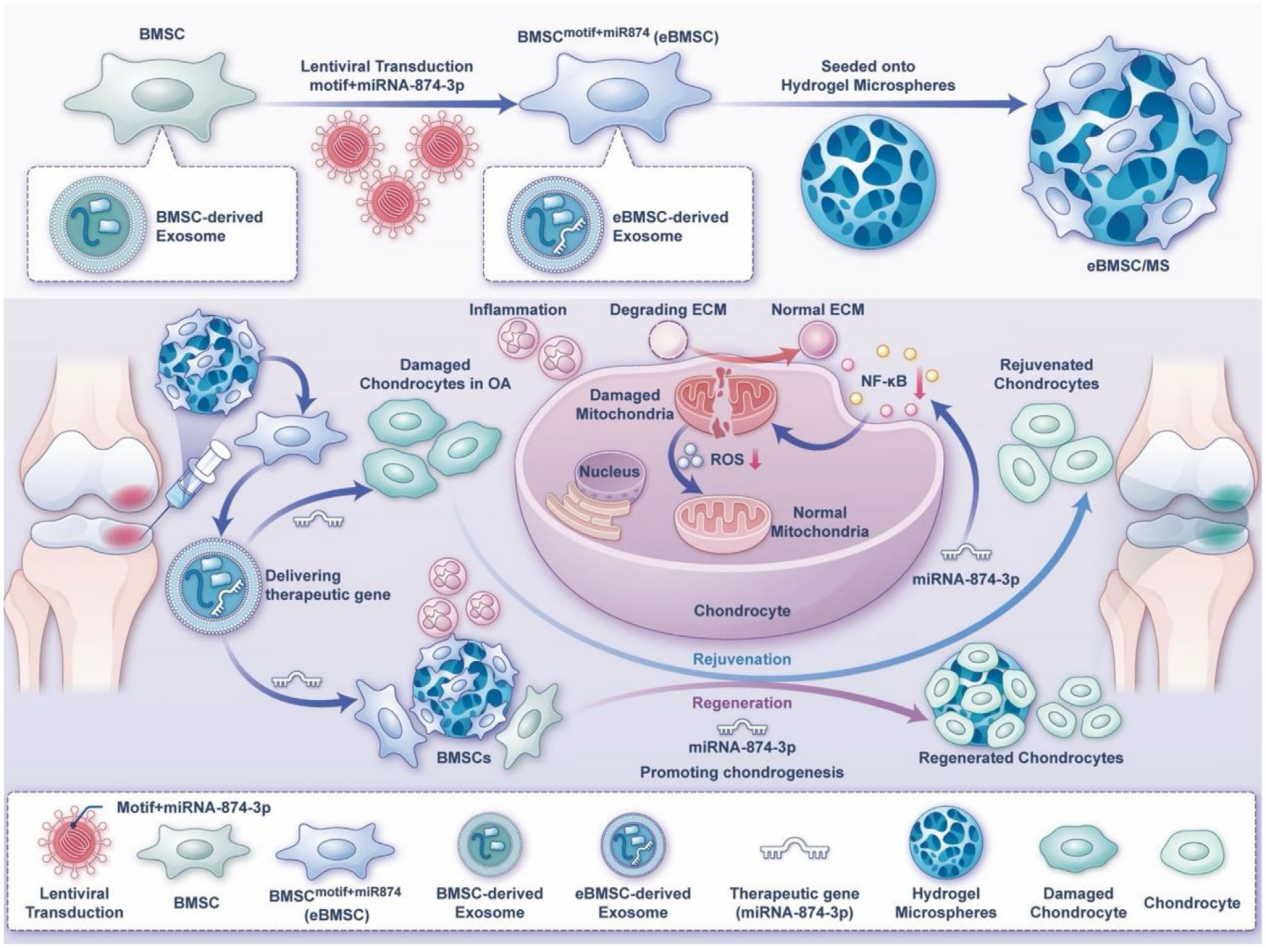
Schematic diagram of BMSC^motif+miR874^/MS synthesis and function.

In this study, we introduced a gene therapy strategy that uses engineered stem cell clusters as a gene delivery platform. Bone marrow‐derived mesenchymal stem cells (BMSCs) were transfected with a fusion gene of miRNA‐874‐3p and an exosome‐targeting motif, successfully generating engineered BMSCs, noted as BMSC^motif+miR874^. These BMSC^motif+miR874^ are characterized by a high abundance of miRNA‐874‐3p in the EVs they secrete. Subsequently, BMSC^motif+miR874^ were loaded onto hydrogel microspheres, creating an injectable hydrogel microsphere system (BMSC^motif+miR874^/MS) for the delivery of engineered stem cell clusters (**Scheme** **1**). In the inflammatory microenvironment and under abnormal mechanical stress associated with OA, the hydrogel microspheres provided a supportive physiological environment for the survival and differentiation of BMSC^motif+miR874^, while also extending the duration of treatment in vivo. This enabled the BMSC^motif+miR874^ in the system to remain active in vivo for a longer period of time, and was able to achieve sustained delivery of the therapeutic gene through the secreted EVs enriched with miRNA‐874‐3p, utilizing the intercellular communication function of EVs. The therapeutic gene, miRNA‐874‐3p, delivered to injured chondrocytes, was capable of enhancing ECM synthesis and effectively reversing the damage caused by inflammation and oxidative stress. Additionally, under inflammatory conditions, BMSC^motif+miR874^ not only exhibited a stronger ability to differentiate into chondrocytes but also promoted the chondrogenesis of other BMSCs. Finally, we established a rat OA model and confirmed that the engineered stem cell clusters enabled the effective delivery of therapeutic genes, alleviated OA progression, and promoted the repair of damaged cartilage. Moreover, this BMSC ^motif+miR874^/MS system, which utilizes EVs enriched with therapeutic genes continuously secreted by living BMSC^motif+miR874^ over a longer period of time, is expected to solve the problem of insufficient persistence of gene delivery in gene therapy, avoiding repeated treatments.

## 2 Results

### 2.1 Preparation and Characterization of BMSC^motif+miR874^/MS

**Figure 1:**
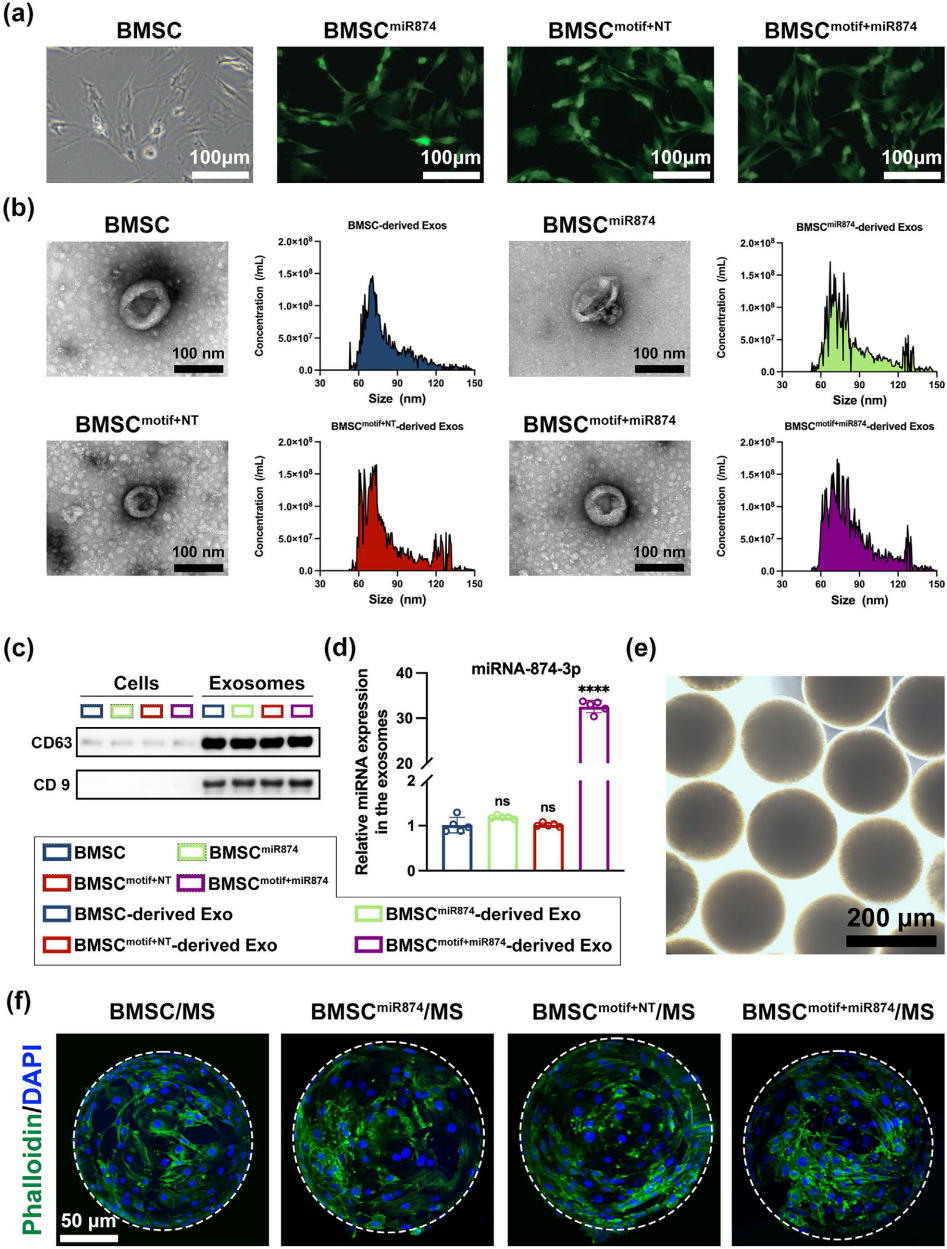
Preparation and characterization of BMSC^motif+miR874^/MS. a) Morphology of BMSCs with or without lentivirus transfection (green) (Scale bar: 100 µm). b) Morphology of each group of BMSC‐derived exosomes detected by TEM (Scale bar: 100 nm) and Nanoparticle tracking analysis of exosomes. c) Western blot analysis of exosome markers CD9 and CD63. d) Relative expression of miRNA‐874‐3p in each group of BMSC‐derived exosomes detected by q‐PCR. e) Microscopic image of GelMA hydrogel microspheres (Scale bar: 200 µm). f) 3D morphology of each group of BMSC/MS detected by CLSM (Scale bar: 50 µm). Data are presented as mean ± SD (*n* = 5, ✱✱✱✱ indicated *p* < 0.0001), with “n” denoting biologically independent experiments. Statistical tests were analyzed by one‐way ANOVA with Tukey's post hoc test.

BMSCs were isolated from the bone marrow of SD rats for genetic engineering. Lentiviruses carrying miRNA‐874‐3p, an exosome‐targeting motif combined with a non‐targeting miRNA (NT), or the motif+miRNA‐874‐3p were used for in vitro transfection of BMSCs to obtain BMSC^miR874^, BMSC^motif+NT^, and BMSC^motif+miR874^, respectively. The successfully transfected BMSCs were selected using puromycin, and the expression of green fluorescent positivity was detected under a fluorescence microscope (**Figure** **1**a). Exosomes derived from each group of BMSCs were then isolated according to the established protocol.^[^ ^24^ ^]^ As shown in Figure 1b, exosomes derived from the four types of BMSCs exhibited a spherical morphology, with most particle sizes ranging from 60 to 150 nm. The results of western blot showed high expression of the exosome surface markers CD9 and CD63 (Figure 1c), indicating the successful isolation of exosomes derived from the four groups of BMSCs.^[^ ^25^ ^]^ Next, real‐time quantitative PCR (q‐PCR) analysis was performed on the four types of BMSC‐derived exosomes to evaluate the expression of miR‐874 in exosomes. As shown in Figure 1d, the expression level of miRNA‐874‐3p was significantly elevated in exosomes derived from BMSC^motif+miR874^. In contrast, exosomes derived from BMSC^miR874^ and BMSC^motif+NT^ showed no significant difference compared to the untransfected BMSC‐derived exosomes. The result demonstrated that the BMSC^motif+miR874^ had the potential to deliver the therapeutic gene miRNA‐874‐3p efficiently by secreting exosomes. In contrast, BMSC^miR874^ obtained by direct transfection of BMSCs with miRNA‐874‐3p gene fragments did not have this ability to deliver therapeutic genes via exosomes.

GelMA hydrogel microspheres with a diameter ≈160 µm (Figure S1b, Supporting Information) were used to construct the BMSC^motif+miR874^/MS system, with the structure shown in Figure S1a (Supporting Information). Served as carriers for BMSC^motif+miR874^, premature degradation of hydrogel microspheres will be unfavorable for the maintenance of BMSC clusters and affects their retention in vivo. To investigate the degradation process of the microspheres, they were immersed in PBS containing 0.1 U mL⁻¹ collagenase II (pH = 7.4) to simulate the physiological microenvironment. As shown in Figure S1d (Supporting Information), during the first two weeks, bright‐field microscopy revealed no significant changes in the morphology or appearance of the microspheres, which remained smooth and spherical. Subsequently, the microspheres gradually became more transparent while maintaining overall integrity, and disordered textures began to appear at the edges. From the fourth week onward, the microspheres started to rupture and collapse at the boundaries, with a progressive increase in transparency. By the sixth week, the microspheres were significantly degraded and destroyed. To further quantify the degradation process, the residual mass of the microspheres was weighed at different time points (Figure S1e, Supporting Information). The above results indicated that the GelMA hydrogel microspheres could basically maintain the spherical shape within 4 weeks, which would help the BMSCs adhered to the microspheres to maintain clusters to keep high survivability and anti‐apoptosis ability. Finally, the BMSCs from each group were seeded onto hydrogel microspheres and cultured in low‐adhesion plates to form the corresponding BMSC/MS systems. Staining of the BMSCs within the systems revealed that all groups of BMSCs exhibited a good growth status, clustering on the surfaces of the hydrogel microspheres (Figure 1f). The above results indicated the successful construction of BMSC/MS system.

### 2.2 Biocompatibility of BMSC^motif+miR874^/MS

First, the activity and proliferation capacity of BMSC^motif+miR874^ were evaluated using CCK‐8 assays. As shown in Figure S2 (Supporting Information), there was no significant difference between original BMSCs and BMSC^motif+miR874^. Further, to confirm the non‐toxicity of BMSC^motif+miR874^/MS‐based gene delivery, we assessed the cell viability of chondrocytes co‐cultured with different groups of BMSC/MS. Results from live/dead cell staining and CCK‐8 assays (Figure S3, Supporting Information) showed that BMSC ^motif+miR874^/MS systems had no significant effect on the cell viability and proliferation of chondrocytes. The results confirmed that the BMSC^motif+miR874^/MS system, through EVs‐mediated therapeutic gene delivery, exhibited good biocompatibility and biosafety.

### 2.3 BMSC^motif+miR874^/MS Protects Chondrocyte Extracellular Matrix

**Figure 2:**
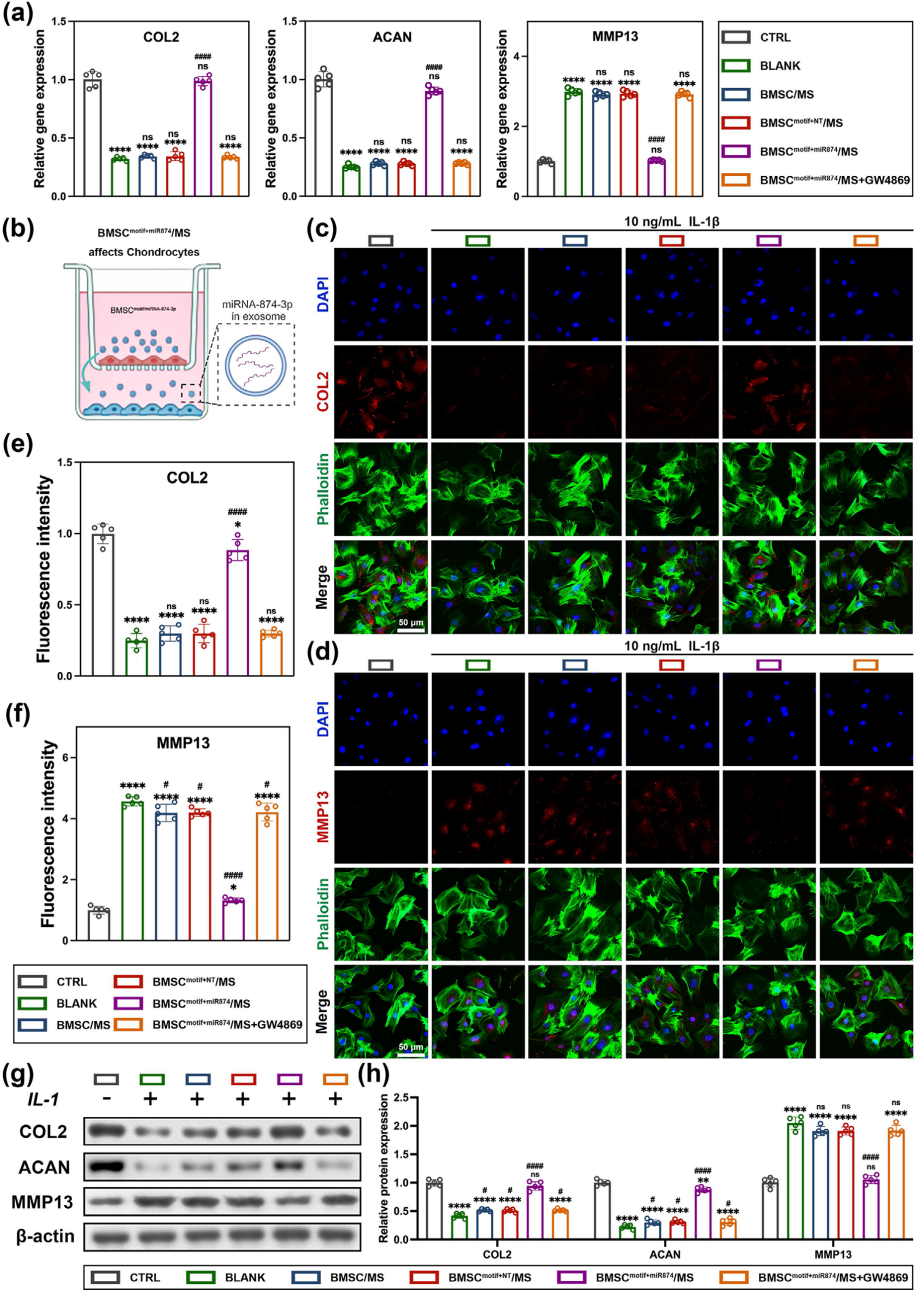
BMSC^motif+miR874^/MS protects chondrocyte extracellular matrix. a) q‐PCR analysis results of COL2, ACAN, and MMP13. b) Schematic illustration of the co‐culture system of chondrocytes. c) Representative immunofluorescence images of COL2 protein (Scale bar: 50 µm). d) Representative immunofluorescence images of MMP13 protein (Scale bar: 50 µm). e) Relative fluorescence intensity quantification of COL2. f) Relative fluorescence intensity quantification of MMP13. g) Western blot results of COL2, ACAN, and MMP13 protein. h) Relative protein expression analysis of Western blot results. Data are presented as mean ± SD (*n* = 5, ✱/✱✱/✱✱✱/✱✱✱✱, #/##/###/#### indicated *p* < 0.05/ *p* < 0.01/ *p* < 0.001/ *p* < 0.0001 in comparison with CTRL and BLANK, respectively), with “n” denoting biologically independent experiments. Statistical tests were analyzed by one‐way ANOVA with Tukey's post hoc test.

The pathogenesis of osteoarthritis is associated with multiple factors, including sustained inflammatory stimulation, oxidative stress, and abnormal mechanical stress, which interfere with the anabolism and catabolism of cartilage matrix by modulating the synthesis of cartilage matrices (e.g., collagen and proteoglycans) and matrix‐degradation‐related enzymes (e.g., matrix metalloproteinases).^[^ ^26^, ^27^, ^28^ ^]^ In this study, we used IL1‐β to model the inflammatory microenvironment in the pathogenesis of osteoarthritis. Next, we constructed a Transwell system as shown in **Figure** **2**b where chondrocytes were seeded in the lower chamber of the Transwell and different BMSC/MS treatment systems were loaded in the upper chamber.

First, the relevant gene expressions in IL‐1β‐stimulated chondrocytes under different treatments were investigated by q‐PCR. As shown in Figure 2a, the gene expression of COL2 and ACAN in chondrocytes treated with BMSC^motif+miR874^/MS was close to that of chondrocytes without IL‐1β stimulation, and was significantly higher than that of the blank group without treatment, the group of normal BMSCs without transfection (BMSC/MS), and the group transfected with meaningless sequences (BMSC^motif+NT^/MS). Whereas, the effect of BMSC^motif+miR874^/MS was significantly reduced after the addition of the exosome secretion inhibitor GW4869, which confirmed that the therapeutic effect of BMSC^motif+miR874^/MS was mediated by exosomes secreted by BMSC^motif+miR874^. Meanwhile, the gene expression changes of MMP13 were in contrast to COL2 and ACAN, and the gene expression of MMP13 in chondrocytes after treatment in the BMSC^motif+miR874^/MS group was significantly reduced, close to that of the CTRL group without inflammatory stimulation.

Immunofluorescence and western blot were conducted to further explore the expression levels of relevant proteins in chondrocytes stimulated by IL‐1β after different groups of treatments (Figure 2c–h). The results of immunofluorescence staining showed that BMSC^motif+miR874^/MS‐treated chondrocytes had the highest COL2 expression in the extracellular matrix, as well as the lowest MMP13 expression, among all inflammatory‐stimulated groups (Figure 2c,d). Moreover, the expression of COL2 and MMP13 in BMSC^motif+miR874^/MS‐treated chondrocytes was close to that of normal chondrocytes, although a small difference still existed from the CTRL group without inflammatory stimulation (Figure 2e,f). The results of western blot were consistent with the previous experiments (Figure 2g,h). The expression of COL2 and ACAN in BMSC^motif+miR874^/MS‐treated IL‐1β‐stimulated chondrocytes was significantly elevated, while the expression of MMP13 was significantly reduced and close to that of normal chondrocytes. And the effect of BMSC^motif+miR874^/MS disappeared after the addition of exosome secretion inhibitor GW4869, which verified the therapeutic effect of EVs secreted in the BMSC^motif+miR874^/MS system on chondrocytes once again. In addition, to further clarify the effect of individual exosome components on chondrocyte therapy, we included an additional group of chondrocytes treated with BMSC‐derived exosomes (without miR874) alone, denoted as EXO, and compared it with the BLANK group as a control for analysis. As demonstrated in Figure S4 (Supporting Information), although the treatment with exosomes alone may exert certain therapeutic effects on chondrocytes, these outcomes failed to attain statistical significance.

Finally, the expression of miRNA‐874‐3p in chondrocytes from each group was measured using q‐PCR. As shown in Figure S5 (Supporting Information), chondrocytes co‐cultured with BMSC^motif+miR874^/MS exhibited the highest levels of miRNA‐874‐3p expression, significantly higher than that of normal chondrocytes. In contrast, the expression of miRNA‐874‐3p in chondrocytes from other inflammation‐stimulated groups, including the BMSC^motif+miR874^/MS+GW4869 group, showed a decrease compared to normal chondrocytes. This indicated that BMSC^motif+miR874^/MS effectively delivered miRNA‐874‐3p to the inflammation‐stimulated chondrocytes via EVs, thereby exerting its therapeutic effect.

Overall, all of these results above showed that the BMSC^motif+miR874^/MS was able to protect the ECM synthesis of inflammation‐stimulated chondrocytes and reduce the synthesis of the catabolic protease MMP13, which protects chondrocytes against inflammatory stimulation from degradation. However, the therapeutic effect of the BMSC^motif+miR874^/MS system was significantly decreased after the addition of an exosome secretion inhibitor, which indicated that the therapeutic effect of BMSC^motif+miR874^/MS was realized through its secreted EVs containing a large amount of miRNA‐874‐3p. In addition, this gene delivery through the exosomes effectively delivered therapeutic genes into chondrocytes, counteracting chondrocyte damage caused by inflammatory stimulation.

### 2.4 BMSC^motif+miR874^/MS Relieves Cellular Oxidative Stress and Protects Chondrocyte Mitochondria

Under sustained inflammatory stimulation, inflammatory mediators such as cytokines (e.g., TNF‐α, IL‐1β) activate intracellular signaling pathways (e.g., NF‐κB, MAPK) through specific receptors, thereby inducing the excessive generation of reactive oxygen species (ROS), which damages mitochondria.^[^ ^29^, ^30^, ^31^ ^]^ Mitochondrial dysfunction leads to the disruption of catabolic processes and intracellular homeostasis in chondrocytes, further exacerbating cellular damage.^[^ ^32^, ^33^ ^]^ Additionally, after mitochondrial damage, redox imbalance results in the accumulation of ROS, forming a vicious cycle. Therefore, mitigating oxidative stress‐induced damage to chondrocytes and restoring mitochondrial function is of critical importance in the treatment of OA.

**Figure 3:**
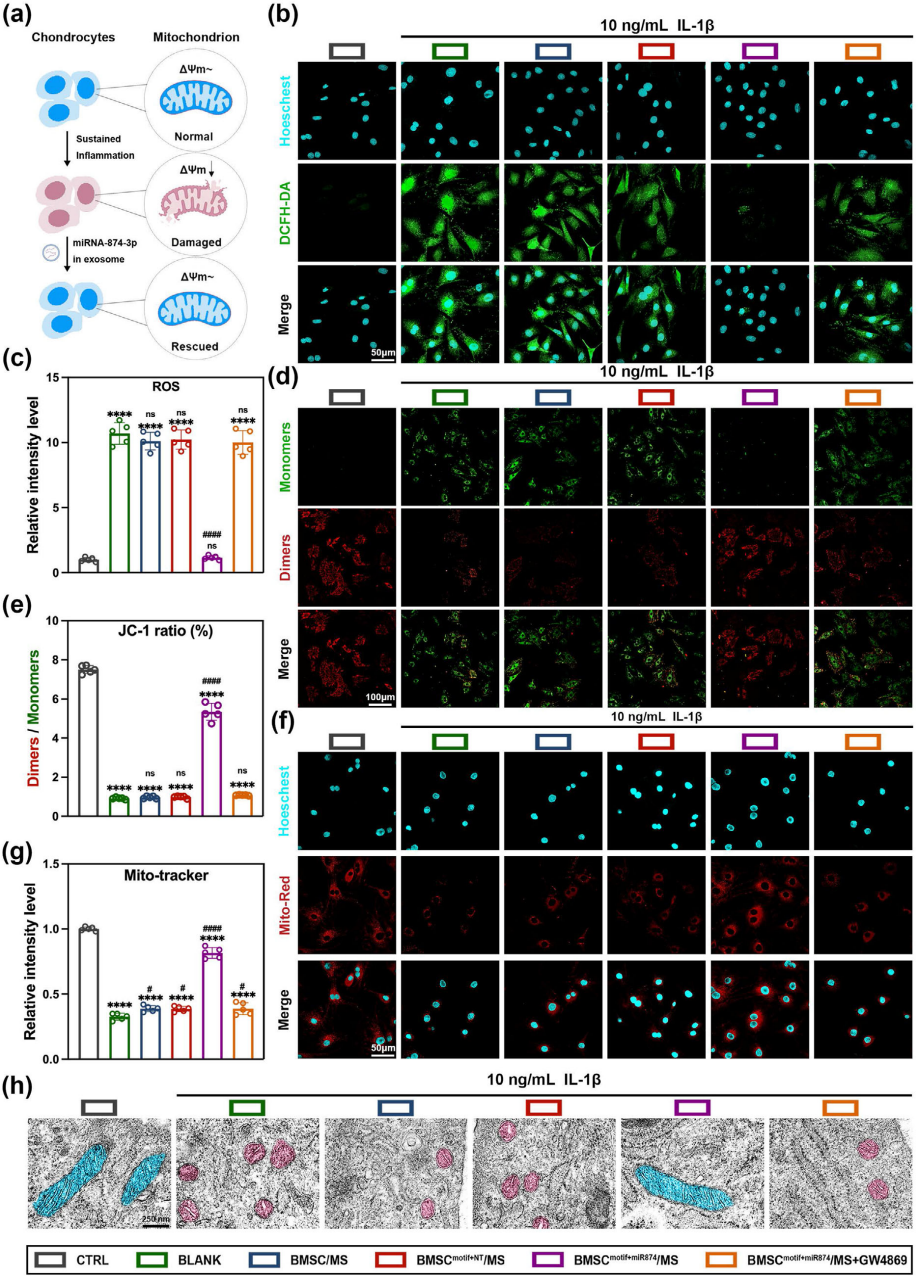
BMSC^motif+miR874^/MS relieves oxidative stress and protects chondrocyte mitochondria. a) Schematic diagram of BMSC^motif+miR874^/MS protecting mitochondrial function and maintaining chondrocyte homeostasis. b) Assessment of ROS generation conducted with DCFH‐DA fluorescent probes (Scale bar: 100 µm). c) Quantification analysis of DCFH‐DA fluorescence intensity. d) Mitochondrial membrane potential detected by JC‐1 staining (Scale bar: 50 µm). e) Quantification analysis of membrane potential by the ratio of red fluorescence to green fluorescence. f) Mitochondrial membrane potential detected by Mito‐tracker Red staining (Scale bar: 50 µm). g) Quantification analysis of Mito‐tracker Red fluorescence intensity. h) TEM images of mitochondria structures in chondrocytes from different treatment groups (Scale bar: 250 nm). Data are presented as mean ± SD (*n* = 5, ✱/✱✱/✱✱✱/✱✱✱✱, #/##/###/#### indicated *p* < 0.05/ *p* < 0.01/ *p *< 0.001/ *p* < 0.0001 in comparison with CTRL and BLANK, respectively), with “n” denoting biologically independent experiments. Statistical tests were analyzed by one‐way ANOVA with Tukey's post hoc test.

In this part of the study, IL‐1β was also used to continuously stimulate chondrocytes to mimic inflammation‐mediated oxidative stress and mitochondrial damage. First, chondrocytes were stained with 2',7'‐dichlorodihydrofluorescein diacetate (DCFH‐DA) probe to assess the intracellular ROS levels, as shown in **Figure** **3**b. ROS accumulation in IL‐1β‐stimulated chondrocytes treated with BMSC^motif+miR874^/MS was significantly reduced compared to the untreated blank group, and was comparable to the levels in normal chondrocytes (Figure 3c). This indicated that after the treatment of BMSC^motif+miR874^/MS, chondrocytes under inflammatory stimulation did not accumulate excessive ROS. However, after the addition of the exosome secretion inhibitor GW4869, the therapeutic effect was lost, and a large accumulation of ROS was observed within the cells. This confirmed that the exosomes derived from BMSC^motif+miR874^, which were capable of delivering miR‐874‐3p, played a crucial role in alleviating cellular oxidative stress.

Next, Mito Tracker Red staining and JC‐1 assays were performed to investigate mitochondrial function in chondrocytes. As shown in Figure 3d, after IL‐1β stimulation, the red fluorescence of JC‐1 aggregates was significantly reduced, while the green fluorescence of JC‐1 monomers increased. Additionally, Mito Tracker Red fluorescence decreased, indicating disruption of the mitochondrial membrane potential and a reduction in mitochondrial numbers (Figure 3f). In chondrocytes co‐cultured with BMSC^motif+miR874^/MS, the JC‐1 red/green ratio (Figure 3e) and the number of mitochondria in chondrocytes (Figure 3g) showed significant recovery compared to the untreated blank group, although they still differed from the levels seen in normal chondrocytes. This suggested that the treatment of BMSC^motif+miR874^/MS resulted in substantial restoration of mitochondrial function, although it did not fully reach the baseline levels of healthy chondrocytes. Finally, mitochondrial morphology in chondrocytes was observed through TEM in Figure 3h. It was found that under inflammatory stimulation (blank group), the mitochondrial matrix structure was disrupted, with evident vacuolation. After the treatment of BMSC^motif+miR874^/MS, mitochondrial damage was significantly reduced, and the mitochondrial matrix structure appeared to be well‐preserved.

During the progress of OA, stress within chondrocytes induced by inflammatory factors and abnormal mechanical stress led to mitochondrial dysfunction, resulting in a decrease in mitochondrial membrane potential and enhanced oxidative stress, which increased ROS release and further exacerbated ROS accumulation.^[^ ^34^ ^]^ Excessive accumulation of ROS, in turn, promoted the intensification of inflammatory responses.^[^ ^35^ ^]^ EVs secreted from BMSC^motif+miR874^/MS, capable of delivering miRNA‐874‐3p, effectively mitigated cellular oxidative stress and protected mitochondria, thereby preventing this vicious cycle. This further demonstrated that BMSC^motif+miR874^/MS had significant potential in the treatment of OA.

### 2.5 Mechanism of BMSC^motif+miR874^/MS in Protecting Chondrocytes in an Inflammatory Environment

**Figure 4:**
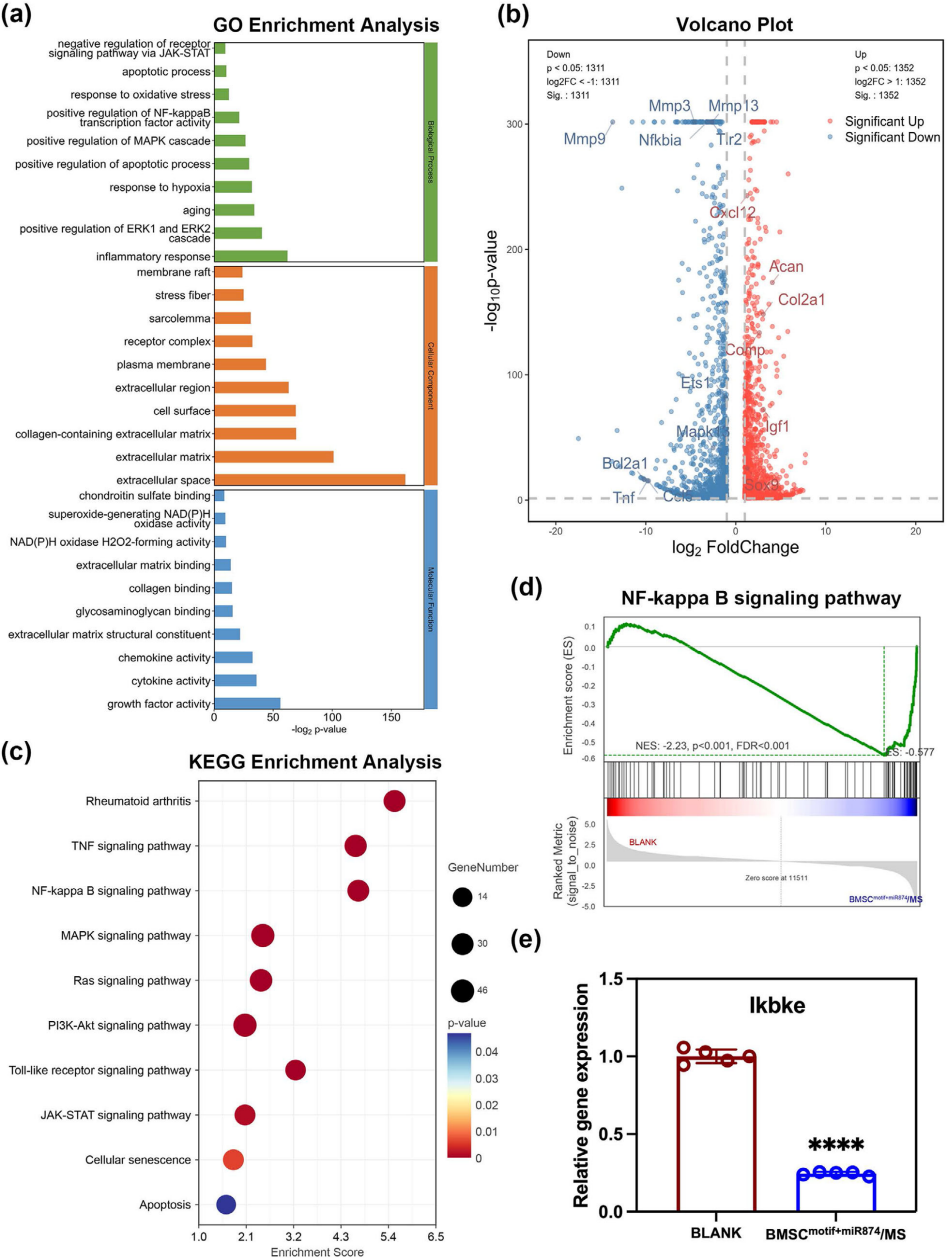
The RNA‐seq analysis of chondrocytes in response to the BMSC^motif+miR874^/MS. a) The genes exhibiting differential expression (DEGs) for Gene Ontology (GO) analysis. b) Volcano plot displaying the differentially expressed genes (fold change ≥ 2 and *p* < 0.05) in chondrocytes co‐cultured with BMSC^motif+miR874^/MS. c) Pathways downregulated in chondrocytes co‐cultured with BMSC^motif+miR874^/MS were analyzed using the KEGG pathway method. d) Gene Set Enrichment Analysis (GSEA) of NF‐κB signaling pathway. e) q‐PCR detection of Ikbke expression in chondrocytes. Data are presented as mean ± SD (*n* = 5, ✱✱✱✱ indicated *p* < 0.0001), with “n” denoting biologically independent experiments. Statistical tests were analyzed by a two‐tailed Student's t‐test.

To investigate the molecular mechanisms underlying the protective effect of the BMSC^motif+miR874^/MS system on chondrocytes under inflammatory stimulation, we performed RNA sequencing on chondrocytes from the BLANK group and the BMSC^motif+miR874^/MS group. Our analysis revealed 2,663 differentially expressed genes (DEGs) shared between the BLANK group and the BMSC^motif+miR874^/MS group, of which 1,352 genes were upregulated and 1,311 genes were downregulated, indicating that the treatment of BMSC^motif+miR874^/MS led to significant changes in gene expression in chondrocytes (**Figure** **4**b).

Next, we performed Gene Ontology (GO) enrichment analysis on these DEGs to systematically categorize their functional roles and enhance our understanding of their biological significance (Figure 4a). The analysis of biological processes (BP) revealed significant differences in inflammation response, aging, and positive regulation of NF‐κB transcription factor activity in chondrocytes co‐cultured with BMSC^motif+miR874^/MS. In the cellular component (CC) analysis, it was found that the collagen‐containing extracellular matrix was significantly increased in chondrocytes co‐cultured with BMSC^motif+miR874^/MS (Figure 4a). Furthermore, the molecular function (MF) analysis revealed significant differences between chondrocytes co‐cultured with BMSC^motif+miR874^/MS and those in the BLANK group in oxidative stress‐related processes, such as NAD(P)H oxidase H_2_O_2_‐forming activity.

We then performed enrichment analysis on Kyoto Encyclopedia of Genes and Genomes (KEGG) pathways. In the BMSC^motif+miR874^/MS group, pathways associated with inflammation and oxidative stress, including TNF, NF‐κB, MAPK, Ras, PI3K‐Akt, Toll‐like receptor, and JAK‐STAT signaling pathways, were all significantly downregulated (Figure 4c). The above signaling pathways were involved in the cellular response to environmental stress, inflammatory stimulation, and oxidative stress. The downregulation of these pathways contributed to the reduction of inflammation levels and oxidative stress, and reduced cellular damage caused by inflammation and oxidative stress, such as mitochondrial damage and ECM degradation, which was important for the protection of chondrocyte function in OA. In addition, pathways related to cellular senescence and apoptosis were also downregulated. This further demonstrated the protective effect of BMSC^motif+miR874^/MS on chondrocytes. Gene Set Enrichment Analysis (GSEA) further confirmed the downregulation of inflammation and oxidative stress‐related pathways in the BMSC^motif+miR874^/MS group (Figure 4d and Figure S8, Supporting Information). In order to further investigate the potential target of the therapeutic gene miR874 delivered by BMSC^motif+miR874^/MS in downregulating inflammation‐related pathways in chondrocytes, we predicted its target through 3′ UTR sequence homology. The Ikbke gene was found to have high credibility as a potential target gene of miR874. The Ikbke gene can directly phosphorylate IκBα, leading to its ubiquitination and degradation, which releases NF‐κB into the nucleus to initiate target gene transcription, playing a crucial regulatory role in the NF‐κB pathway. Next, the expression of the Ikbke gene in chondrocytes from the BLANK and BMSC^motif+miR874^/MS groups was assessed by q‐PCR (Figure 4e). As predicted, the expression of the Ikbke gene in chondrocytes from the BMSC^motif+miR874^/MS group was significantly reduced, indicating that Ikbke is one of the direct targets of miRNA‐874‐3p in chondrocytes. BMSC^motif+miR874^/MS delivered the therapeutic gene to chondrocytes and then downregulated the activation of NF‐κB signaling by suppressing Ikbke, thereby exerting a potential protective effect on chondrocytes.

Therefore, these results suggested that chondrocytes co‐cultured with BMSC^motif+miR874^/MS were able to effectively respond to inflammatory stimulation. By downregulating inflammation‐ and stress‐related signaling pathways, such as NF‐κB and Toll‐like receptor signaling pathways, they reduced the damage caused by inflammation and oxidative stress, thereby protecting chondrocyte function.

### 2.6 BMSC^motif+miR874^/MS Promotes Chondrogenesis in an Inflammatory Microenvironment

The therapeutic gene we applied, miRNA‐874‐3p, had been shown to promote chondrogenesis.^[^ ^19^ ^]^ Additionally, our previous experiments had confirmed that miRNA‐874‐3p could downregulate inflammatory pathways, such as NF‐κB, in chondrocytes. We hypothesized that the therapeutic gene (miRNA‐874‐3p) delivered by BMSC^motif+miR874^/MS could enhance the chondrogenic differentiation of stem cells in the inflammation microenvironment.

**Figure 5:**
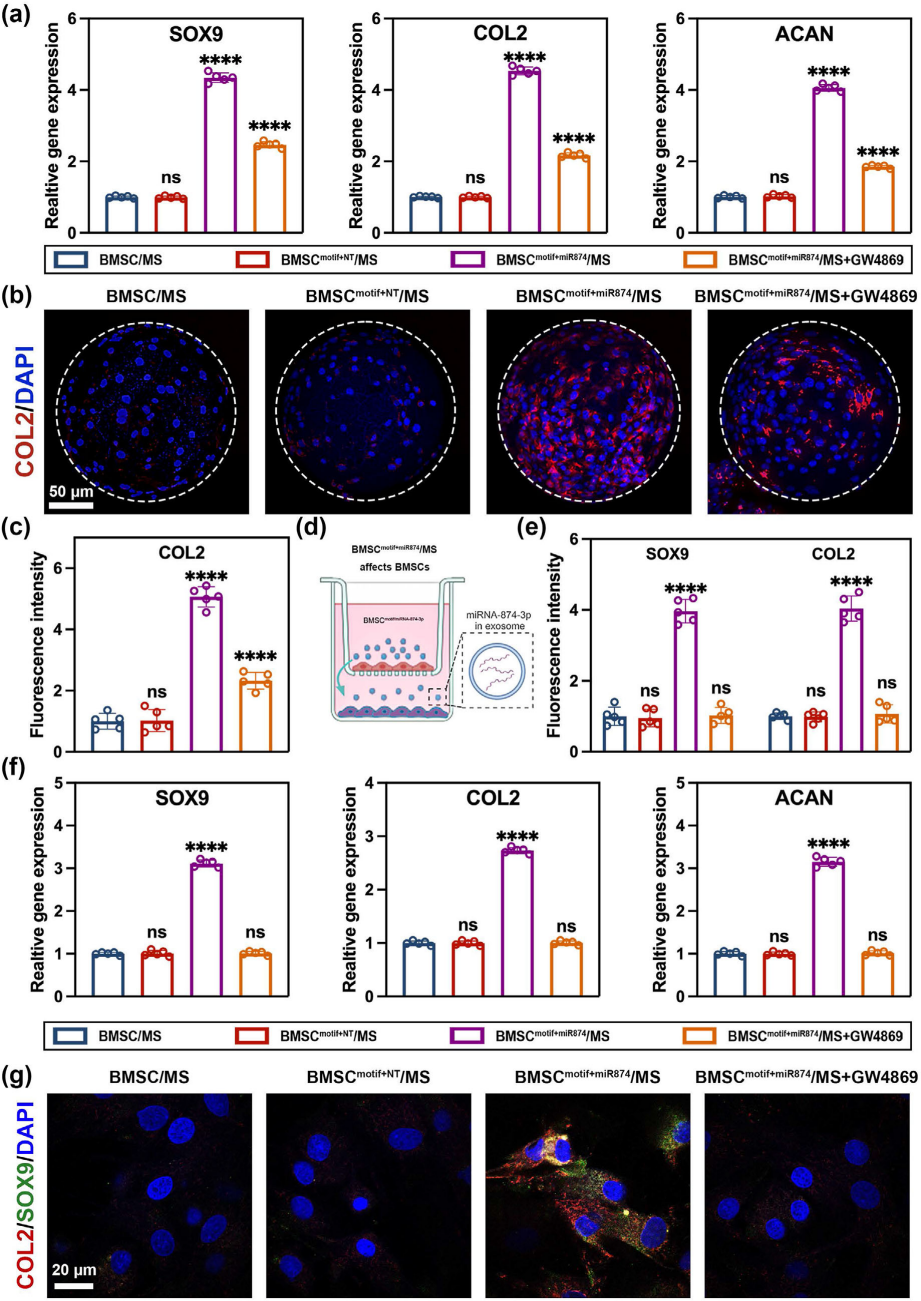
BMSC^motif+miR874^/MS promotes chondrogenic differentiation of BMSCs in inflammatory microenvironment. a) q‐PCR analysis results of SOX9, COL2, and ACAN expression from different BMSC clusters. b) Protein of COL2 (red) expression from different BMSC clusters was visualized by IF staining (Scale bar: 50 µm). c) Quantification analysis of COL2 fluorescence intensity according to different BMSC clusters. d) Schematic illustration of the co‐culture system of BMSCs. e) Quantification analysis of COL2 (red) and SOX9 (green) fluorescence intensity according to BMSCs co‐cultured with different BMSC clusters. f) q‐PCR analysis results of SOX9, COL2, and ACAN expression from BMSCs co‐cultured with different BMSC clusters. g) Protein of COL2 (red) and SOX9 (green) expression from BMSCs co‐cultured with different BMSC clusters was visualized by IF staining (Scale bar: 20 µm). Data are presented as mean ± SD (*n* = 5, ✱✱✱✱ indicated *p* < 0.0001 in comparison with BMSC/MS), with “n” denoting biologically independent experiments. Statistical tests were analyzed by one‐way ANOVA with Tukey's post hoc test.

First, we investigated the chondrogenic differentiation of BMSCs clustered on hydrogel microspheres after 14 days of chondrogenic differentiation‐inducing culture. The BMSC^motif+miR874^/MS group exhibited the highest transcriptional expression of chondrogenic markers (SOX9, COL2, and ACAN) (**Figure** **5**a). Furthermore, immunofluorescence staining was used to evaluate the expression of chondrogenic markers (COL2) at the protein level (Figure 5b). Similarly, the BMSC^motif+miR874^/MS group showed the highest levels of expression (Figure 5c). Notably, upon the addition of the exosome secretion inhibitor GW4869, the expression levels of chondrogenic markers in BMSCs^motif+miR874^ decreased but remained significantly higher than those in non‐transfected BMSCs. This could be attributed to the fact that during the clustering of BMSCs^motif+miR874^ onto hydrogel microspheres to construct the BMSC^motif+miR874^/MS system, a certain degree of therapeutic gene delivery among BMSCs^motif+miR874^ had already been achieved via EVs. Next, we used Transwell assays to further investigate the effect of BMSC^motif+miR874^/MS on the chondrogenic differentiation of other BMSCs after 14 days of culture (Figure 5d). As shown in Figure 5f, BMSCs co‐cultured with BMSC^motif+miR874^/MS showed the highest transcriptional expression of chondrogenic markers (SOX9, COL2, and ACAN). The immunofluorescence staining results were consistent with the q‐PCR findings, where BMSCs in the BMSC^motif+miR874^/MS group showed the highest protein expression of chondrogenic markers (SOX9 and COL2) (Figure 5e,g). In the BMSC^motif+miR874^/MS +GW4869 group, the expression of chondrogenic markers in BMSCs was significantly decreased compared to the group without the exosome secretion inhibitor, indicating that inhibition of exosome secretion hindered the delivery of the therapeutic gene to other BMSCs. These results indicated that the delivery of miRNA‐874‐3p via EVs by BMSC^motif+miR874^/MS not only promoted the chondrogenic differentiation of BMSCs clustered on hydrogel microspheres in an inflammatory microenvironment but also enhanced chondrogenesis in the surrounding BMSCs within the microenvironment.

### 2.7 Therapeutic Effects of BMSC^motif+miR874^/MS in Rat OA Model

**Figure 6:**
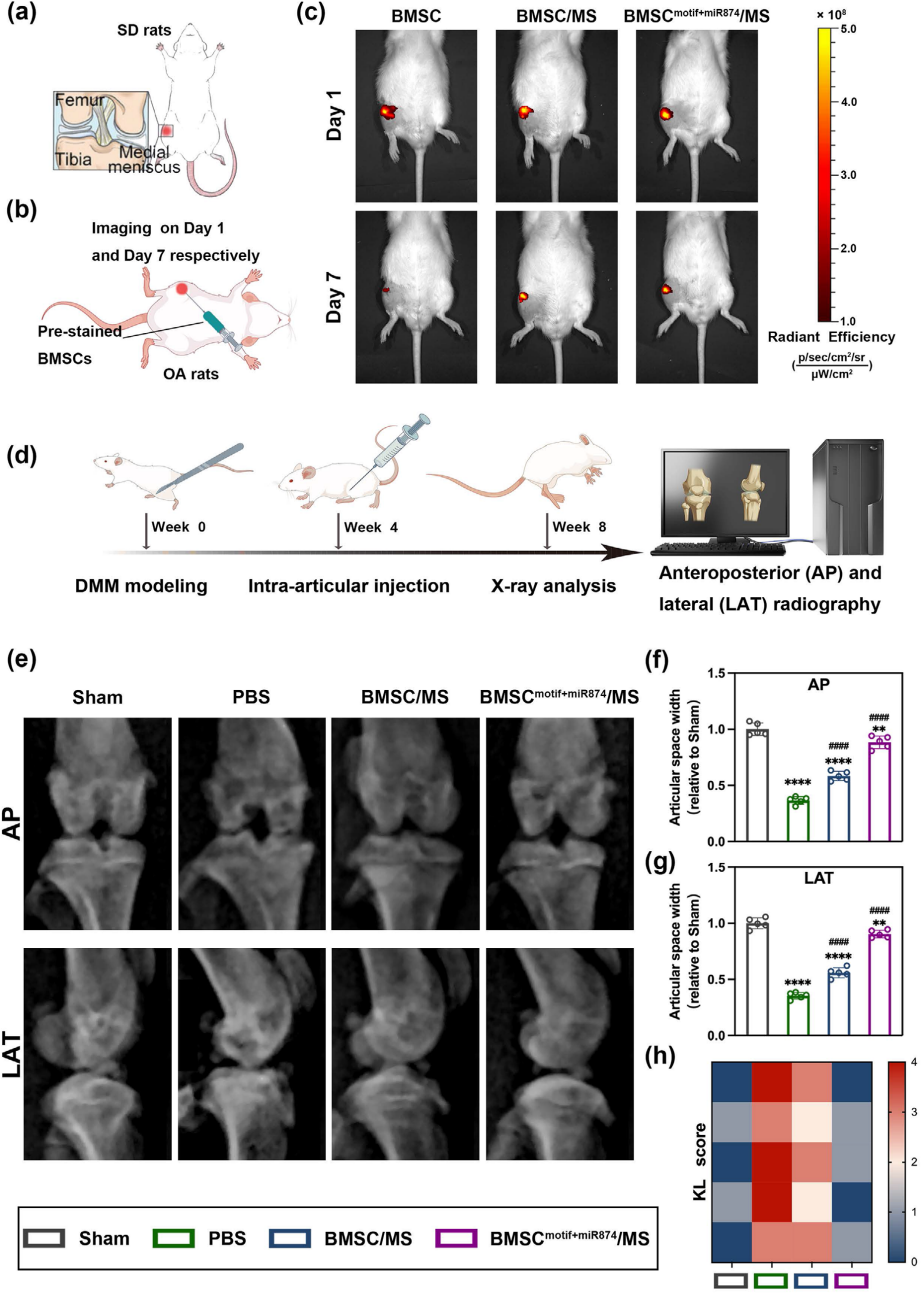
In vivo retention assessment of BMSC^motif+miR874^/MS and radiographic assessment of therapeutic effects in rat OA model. a) Study design of rat OA model. b) Schematic illustration of in vivo retention assessment in rat OA model. c) Luminescence images of rats after intra‐articular injection of Cy7‐labeled BMSCs. d) Schematic diagram of in vivo experiment process. e) Radiographic assessment of the knee joint. f) Articular space width assessment according to the anteroposterior view of the knee joint. g) Articular space width assessment according to the lateral view of the knee joint. h) KL OA score assessment according to X‐ray images. Data are presented as mean ± SD (*n* = 5, ✱/✱✱/✱✱✱/✱✱✱✱, #/##/###/#### indicated *p* < 0.05/ *p* < 0.01/ *p* < 0.001/ *p* < 0.0001 in comparison with the Sham and PBS groups, respectively), with “n” denoting biologically independent experiments. Statistical tests were analyzed by one‐way ANOVA with Tukey's post hoc test.

To evaluate the therapeutic effects of BMSC^motif+miR874^/MS in osteoarthritis, we performed in vivo studies using the rat OA model. The model was induced by destabilization of the medial meniscus (DMM) surgery, which effectively simulated an in vivo inflammatory environment and provided good reproducibility and reliability^[^ ^36^ ^]^ (**Figure** **6**a).

First, we investigated the distribution of Cy7‐labeled BMSCs in the joint cavities of OA rats (Figure 6b). Imaging was performed on Day 1 and 7 after injection, respectively, to observe the retention time of BMSCs in the joint cavity across different groups. As shown in Figure 6c, fluorescence signals were clearly localized in the rat knee joints on the first day after intra‐articular injection. On day 7, the fluorescence signal of the individually injected BMSCs had mostly dissipated. In contrast, BMSCs clustered on hydrogel microspheres (BMSC/MS and BMSC^motif+miR874^/MS) still exhibited strong signal expression, indicating long‐term retention of BMSC/MS and BMSC^motif+miR874^/MS in cartilage. As shown in Figure S9 (Supporting Information), the statistical analysis of the attenuation rates further confirmed that the retention time in vivo of BMSCs aggregated into clusters in both BMSC/MS and BMSC^motif+miR874^/MS groups was significantly higher than that of the fragmented distribution of BMSCs. In addition, the attenuation rate in the BMSC^motif+miR874^/MS group appeared to be slightly lower than that in the BMSC/MS group, but this difference was not statistically significant. The above results indicated that hydrogel microspheres could effectively protect exogenous BMSCs in the OA environment and prolong their retention in vivo for a longer therapeutic period.

Next, 4 weeks after the intra‐articular injection of each group of therapeutics, rats in each group were subjected to anteroposterior and lateral radiography to assess the progression of OA by observing the joint space width (Figure 6d). As shown in Figure 6e–g, compared with the Sham group, the joint space widths were significantly reduced in the PBS group, with obvious subchondral bone erosion and a large number of bone spurs formed, proving the successful establishment of the DMM model. In contrast, the joint space widths in the BMSC^motif+miR874^/MS group were significantly increased compared to the PBS group and were closest to the level of the Sham group. The BMSC/MS group, on the other hand, also showed a slight improvement compared to the PBS group, but still had a large gap compared to the Sham group. The KL score was used to further assess the progress of OA.^[^ ^37^ ^]^ The result confirmed that the OA progression was severe in the PBS group, with the BMSC/MS group showing only slight improvement compared to the PBS group (Figure 6h). In contrast, the BMSC^motif+miR874^/MS group exhibited overall better joint condition, which was close to the Sham group. The evaluation of X‐rays indicated that the treatment of BMSC^motif+miR874^/MS protected against the degeneration of joint structure in OA and may have the potential to alleviate joint damage.

**Figure 7:**
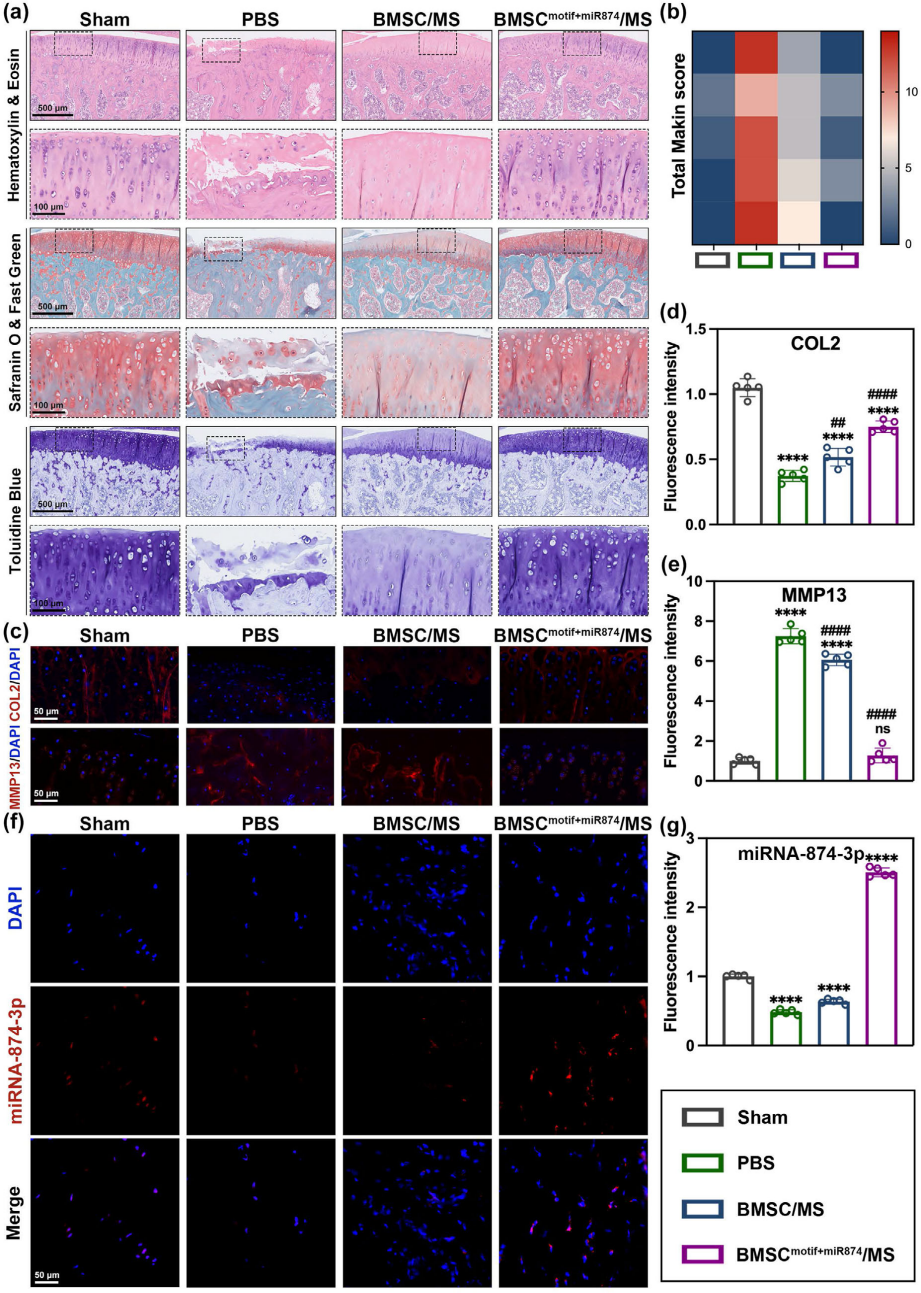
Histological evaluation of the therapeutic effects of BMSC^motif+miR874^/MS in OA rat model. a) H&E, Safranin O‐fast green, and Toluidine Blue staining from each group (Scale bar: down (enlarged area), 100 µm; up, 500 µm). b) Total Mankin score assessment. c) IFC staining of COL2 and MMP13 from each group (Scale bar: 50 µm). d) Quantification of relative COL2 expression. e) Quantification of relative MMP13 expression. f) Evaluation of miRNA‐874‐3p by fluorescence in situ hybridization (Scale bar: 50 µm). g) Quantification of relative miRNA‐874‐3p expression. Data are presented as mean ± SD (*n* = 5, ✱/✱✱/✱✱✱/✱✱✱✱, #/##/###/#### indicated *p* < 0.05/ *p* < 0.01/ *p* < 0.001/ *p* < 0.0001 in comparison with the Sham and PBS groups, respectively), with “n” denoting biologically independent experiments. Statistical tests were analyzed by one‐way ANOVA with Tukey's post hoc test.

Next, histological analysis of rat knee joint samples was performed through H&E, Safranin O‐Fast Green, and Toluidine Blue staining (**Figure** **7**a). In the PBS group, significant cartilage damage was observed, including severe cartilage erosion and extensive loss of glycosaminoglycans (GAGs). In the BMSC/MS group, the degree of cartilage erosion was milder, but there was still noticeable loss of GAGs. In contrast, the cartilage structure and morphology in the BMSC^motif+miR874^/MS group were nearly intact, with GAGs content maintained at high levels, similar to the Sham group. As demonstrated by the Mankin score (Figure 7b), the degenerative changes of cartilage were significantly alleviated in the BMSC^motif+miR874^/MS group. Immunofluorescence analysis was performed to evaluate the protein levels of COL2 and MMP13. As shown in Figure 7c–e, the fluorescence intensity of COL2 in articular cartilage decreased in both the PBS and BMSC/MS groups compared to the Sham group. In contrast, the fluorescence intensity of COL2 in the BMSC^motif+miR874^/MS group was significantly higher than that in the PBS group and closer to the level observed in the Sham group. Additionally, the treatment of BMSC^motif+miR874^/MS significantly reduced MMP13 expression in articular cartilage, with the fluorescence intensity of MMP13 showing no significant difference from that in the Sham group. These results indicated that BMSC^motif+miR874^/MS could inhibit cartilage degradation in OA and had the potential to achieve repair of mild cartilage damage and prevent progression to advanced stages.

Additionally, fluorescence in situ hybridization (FISH) was conducted to detect the expression of miRNA‐874‐3p in cartilage, verifying the delivery of the therapeutic gene by BMSC^motif+miR874^/MS (Figure 7f). Fluorescence intensity analysis revealed that miRNA‐874‐3p expression in the PBS and BMSC/MS groups was significantly lower compared to the Sham group (Figure 7g). In contrast, the BMSC^motif+miR874^/MS group exhibited significantly increased miRNA‐874‐3p expression levels, which were notably higher than those in the Sham group, confirming that BMSC^motif+miR874^/MS successfully delivered the therapeutic gene to the articular cartilage.

Finally, we evaluated the in vivo biocompatibility of BMSC^motif+miR874^/MS. H&E staining of major organs showed no damage in the BMSC^motif+miR874^/MS group compared to the Sham group (Figure S10, Supporting Information). These findings strongly demonstrated the biosafety of the therapeutic gene delivery approach mediated by BMSC^motif+miR874^/MS through EVs.

## 3 Discussion

Osteoarthritis, as a chronic degenerative disease, still lacks a thorough and effective therapeutic regimen, and intervention with non‐surgical therapies in the early stages of disease progression is an urgent clinical need. In this study, we propose a novel EVs‐mediated sustained gene delivery system, BMSC^motif+miR874^/MS. This system combines engineered BMSCs with hydrogel microspheres to form engineered stem cell clusters, which utilize the continuous secretion of EVs by living BMSC^motif+miR874^ for intercellular communication to achieve sustained therapeutic gene delivery. Our findings demonstrate that BMSC^motif+miR874^/MS effectively mitigates cartilage degradation by restoring chondrocytes and promoting chondrogenesis in inflammatory and oxidative stress microenvironments, providing a promising strategy for OA treatment.

The key innovation of this work lies in the integration of EVs‐mediated miRNA delivery with a hydrogel‐based engineered stem cell cluster retention platform. By fusing miRNA‐874‐3p with an exosome‐targeting motif, we engineered BMSCs to secrete exosomes enriched with the therapeutic gene, miRNA‐874‐3p. This approach overcomes the limitations of conventional gene delivery methods, such as the rapid degradation and low transfection efficiency of free miRNAs or the insufficient in vivo duration of effect of engineered EVs alone. Importantly, GelMA hydrogel microspheres not only provide a protective niche for BMSCs to resist inflammatory stimuli but also promote their aggregation into clusters, enhancing their resistance to mechanical stress. This reduces cell damage and loss, thereby extending their retention time in vivo and ensuring the long‐term secretion of engineered EVs.

Mechanistically, miRNA‐874‐3p delivered from BMSC^motif+miR874^/MS exhibited a dual therapeutic effect: 1) it inhibited inflammatory signaling pathways such as NF‐κB in chondrocytes, thereby reducing inflammation and oxidative stress‐induced chondrocyte damage, and 2) it promoted chondrogenic differentiation of BMSCs even under inflammatory conditions. These results are consistent with previous studies that emphasized the role of miR‐874‐3p in inhibiting matrix metalloproteinases and enhancing cartilage repair.^[^ ^19^, ^20^ ^]^ The use of GW4869 to inhibit exosome secretion unequivocally validated the indispensability of EVs abundant in miRNA‐874‐3p in mediating these effects. Our RNA‐seq analyses further revealed broader regulatory impacts, including downregulation of senescence‐associated pathways and restoration of mitochondrial function, which collectively promote chondrocyte rejuvenation. In addition, our preliminary exploration of miRNA‐874‐3p's targets in chondrocytes revealed that the expression of Ikbke—a key gene in the NF‐κB pathway—was significantly downregulated in the BMSC^motif+miR874^/MS group. Ikbke directly phosphorylates IκBα, triggering its ubiquitination and degradation, which subsequently releases NF‐κB subunits for nuclear translocation and activation of downstream pro‐inflammatory genes.^[^ ^38^ ^]^ The observed reduction in Ikbke expression suggests that miRNA‐874‐3p may directly target this gene, thereby inhibiting NF‐κB pathway activation and contributing to the alleviation of OA‐related chondrocyte dysfunction.

In vivo, the BMSC^motif+miR874^/MS system demonstrated superior therapeutic outcomes. The hydrogel microspheres significantly prolonged BMSC retention in joint cavity, enabling sustained miRNA‐874‐3p release and resulting in reduced cartilage degradation, improved joint space width, and enhanced ECM preservation. The FISH assay confirmed that BMSC^motif+miR874^/MS achieved effective delivery of therapeutic genes in vivo, further validating the important protective role of miRNA‐874‐3p on articular cartilage during OA progression. Notably, the system's biosafety was confirmed by the absence of organ toxicity, underscoring its translational potential.

Despite these advances, limitations must be acknowledged. First, while the rat DMM model effectively mimics OA progression, human joints exhibit more complex biomechanical and inflammatory environments. Future studies should validate the system on larger animal models or human‐derived articular organoid platforms for long‐term biosafety evaluation. Second, BMSC^motif+miR874^‐derived EVs themselves require further study, especially a comprehensive analysis of their differences with original BMSC‐derived EVs beyond the overexpression of miRNA‐874‐3p and their potential interactions with non‐cartilage tissues. Moreover, optimizing the hydrogel composition to enhance mechanical resilience under dynamic joint loading could improve clinical applicability.

## 4 Conclusion

Gene therapy, as a non‐surgical treatment approach, offers precise regulation of gene expression and long‐lasting therapeutic effects, making it a promising strategy for OA treatment. However, the safe and effective delivery of therapeutic genes has been a major challenge in gene therapy. In this study, we combined the therapeutic gene sequence with an exosome‐targeting motif to engineered BMSCs capable of safely and effectively delivering therapeutic genes via EVs. Furthermore, we developed the BMSC^motif+miR874^/MS system, which effectively protected exogenous BMSCs under the abnormal mechanical stress and inflammatory microenvironment present in OA joints. This ensured the long‐term and safe delivery of therapeutic genes via EVs in vivo. In addition, miRNA‐874‐3p, selected as the therapeutic gene in this study, demonstrated the ability to rejuvenate chondrocytes in the inflammatory and oxidative stress microenvironment of OA, effectively protecting chondrocytes. Furthermore, it promoted the chondrogenic differentiation of BMSCs under inflammatory conditions, facilitating cartilage regeneration and the repair of early cartilage damage in OA. These effects contributed to achieving effective treatment for OA. Therefore, the BMSC^motif+miR874^/MS system proposed in this study holds promising potential for the treatment of OA. Moreover, this EVs‐mediated therapeutic gene delivery approach may also be applicable to the treatment of other diseases, offering a novel option for gene therapy.

## 5 Experimental Section

### Ethics

The experimental animals were purchased from Jiagan Biotechnology Co., Ltd. and maintained in a controlled environment with a 12 h light/dark cycle. The animals had free access to food and water. The animal experiments were approved by Jiagan Experimental Animal Management Ethics Committee (Approval No.: JGLL‐20231231).

### Statistical Analysis

All experiments were performed with biological replicates, and each experimental iteration was independently repeated at least five times. Representative data are shown here. Quantitative data were expressed as mean ± standard deviation. Subsequent comparative analyses were performed using GraphPad Prism 9 software (GraphPad Software, Inc., USA). For comparisons between two groups, a two‐tailed Student's t‐test was executed, while multiple comparisons were analyzed using one‐way ANOVA with Tukey's post hoc test. *p* < 0.05 were considered statistically significant.

The experimental details, including the synthesis and characterization of BMSC^motif+miR874^/MS, as well as the in vitro and in vivo experiments, are provided in the Supporting Information.

## Conflict of Interest

The authors declare no conflict of interest.

## Author Contributions

Y.W., Y.F., and F.H. contributed equally to this work.

## Supporting information



Supporting Information

## Data Availability

The data that support the findings of this study are available from the corresponding author upon reasonable request.
