# Supplementary material for: Engineered Stem Cell Clusters for Extracellular Vesicles‐Mediated Gene Delivery to Rejuvenate Chondrocytes and Facilitate Chondrogenesis in Osteoarthritis Therapy
Source: Adv Sci (Weinh). 2025 Apr 25;12(25):2500964. doi: 10.1002/advs.202500964 (PMC12224965; doi:10.1002/advs.202500964)

**Engineered Stem Cell Clusters for Extracellular Vesicles-Mediated Gene Delivery to Rejuvenate Chondrocytes and Facilitate Chondrogenesis in Osteoarthritis Therapy**

**Yuezhou Wu** ^a, †^**, Yubo Feng** ^d, †^**, Fei Hu** ^a, †^**, Xu Zheng** ^a^**, Yurun Ding** ^a^**, Xuesong Liu** ^c,^ ***, Shicheng Huo** ^b,^ ***, Zhuocheng Lyu** ^a,^ *

^a^ Department of Bone and Joint Surgery, Department of Orthopedics, Renji Hospital, School of Medicine, Shanghai Jiaotong University, Shanghai, China

^b^ Department of Orthopedic Surgery, Spine Center, Changzheng Hospital, Navy Medical University, Shanghai, China.

^c^ Department of Ultrasound, Renji Hospital, School of Medicine, Shanghai Jiao Tong University, Shanghai, China.

^d^ Department of Spine Surgery, Department of Orthopedics, Renji Hospital, School of Medicine, Shanghai Jiaotong University, Shanghai China.

* Corresponding authors:

E-mail: Xuesong Liu (liuxuesong@renji.com), Shicheng Huo (waznxyz@126.com), Zhuocheng Lyu ([lzc2015@sjtu.edu.cn](mailto:lzc2015@sjtu.edu.cn))

^†^ These authors contributed equally to this work.

**Experimental Section**

***Construction of Engineered BMSCs:***

The empty lentiviral vector pXMIRX (SBI, USA) was linearized using BamH I and EcoR I. The annealed oligonucleotide duplex encoding miRNA-874-3p was ligated into the linearized vector, and the ligation product was transformed into Escherichia coli. Plasmids were extracted from successful colonies. For virus packaging, LentiX 293T cells were transfected with the packaging plasmids psPAX2, pMD2.G, and pXMIRX-miR-874-3p in a 12 µg: 9 µg: 3 µg ratio, using Lipofectamine 2000. After 12 hours, the medium was replaced, and supernatants were collected on days 1 and 2. On day 4, the supernatants were centrifuged, filtered, and concentrated using a 100 kDa cutoff filter. The concentrated virus was stored at -80°C. Rat primary BMSCs were seeded in 6-well plates and cultured until 60-70% confluence. The lentiviral stock was thawed, diluted in DMEM/F12, and supplemented with polybrene (1/1000). The virus was added to the cells and the medium was replaced with fresh DMEM/F12 after 8 hours. After 48 hours, transfection efficiency was assessed by GFP expression under a fluorescence microscope, finally obtaining BMSC^motif+miR874^. Similarly, replacing the gene sequence of miRNA-874-3p with that of a non-targeting miRNA to obtain BMSC^motif+NT^. In contrast, replacing pXMIRX-miR-874-3p with miR-874-3p for subsequent steps resulted in BMSC^miR874^.

***Isolation and Characterization of Engineered BMSCs-derived Exosomes:***

When the engineered BMSCs in each group reached 80%-90% confluence, they were cultured in serum-free medium for 24-48 hours to promote exosome secretion. After cultivation, the culture medium was collected and centrifuged at 300g for 10-15 minutes to remove cells and larger particles, retaining the supernatant. The supernatant was then further centrifuged at 10,000g for 30 minutes to eliminate cell debris and larger particles. Next, exosomes were isolated through ultracentrifugation. The cleared supernatant was subjected to centrifugation at 100,000g for 1 hour at 4°C, and the resulting pellet was collected as exosomes. Transmission electron microscopy (TEM) was used to observe the typical spherical structure of the exosomes, and nanoparticle size analysis was performed to determine their particle size. Exosome marker proteins (CD9 and CD63) were detected by western blot to validate the extracted exosomes. Finally, the expression levels of miR-874 in exosomes derived from BMSCs in each group were measured by quantitative PCR (qPCR), with the primer sequences used shown in Table S1.

**Table S1** Primers used in this experiment.

| Gene and primer direction | Primer sequence |
| --- | --- |
| rno-miR-874-3p |  |
| Forward | CTGCCCTGGCCCGAGG |
| Reverse | CAGTGCAGGGTCCGAGGTAT |
| cel-miR-39 |  |
| Forward | AGCCCGTCACCTGGTGTAAATC |
| Reverse | CAGTGCAGGGTCCGAGGTAT |

***Fabrication and Characterization of GelMA Hydrogel Microspheres:***

10 wt% gelatin (Sigma, US) was reacted with methacrylic anhydride (Sigma, US) in carbonate buffer (pH 9.0) for 3 hours. After the reaction, the product was dialyzed for 2 days and then lyophilized to obtain GelMA hydrogels. Next, GelMA and photoinitiator LAP were dissolved in deionized water to prepare a solution. The oil phase solution was prepared by mixing paraffin oil with Span80. The flow rate ratio of the aqueous phase to the oil phase in the microfluidic device was adjusted at 60°C, and the cross-linking reaction was performed under UV light to obtain hydrogel microspheres. The particle size and general morphology of the GelMA hydrogel microspheres were investigated and analyzed by bright field microscopy (Zeiss, Germany). The pore size and surface morphology of lyophilized microspheres and stem were observed by SEM (Hitachi, Japan). To study the degradation of aqueous GelMA gel microspheres, the microspheres were immersed in a degradation solution consisting of PBS (pH=7.4) and 0.1 U mL^-1^ collagenase II and placed in a shaker (37°C, 80 rpm) to simulate physiological conditions. Images were taken and weights were recorded at specific time points. Moreover, rheological evaluation of GelMA hydrogels was performed by rheometer (MARS 60, USA).

***Construction and Characterization of the BMSC^motif+miR874^/MS:***

First, hydrogel microspheres were seeded into a low-adhesion culture plate. Then, 2 × 10⁴ cells per mL of engineered BMSCs were added to the culture plate containing the hydrogel microspheres and co-cultured for 24 hours to obtain hydrogel microspheres with BMSCs adhering to their surface (BMSC/MS). After fixing the cells with 4% paraformaldehyde, the BMSC/MS system was stained with Actin-Tracker Green-488 (Beyotime, China) for 30 minutes, followed by DAPI staining for 5 minutes. Three-dimensional imaging of the BMSC/MS system in each group was performed using a confocal laser scanning microscope (CLSM, Olympus, Japan) to observe the adhesion and growth of BMSCs on the surface of the hydrogel microspheres.

***In vitro Biocompatibility Assessment:***

Each group of BMSC/MS was injected into the upper chamber of a transwell, which was then inserted into a culture plate seeded with chondrocytes for co-culture. After 1 and 3 days of co-culture, chondrocytes in the co-culture system were stained using a Calcein/PI Cell Viability and Cytotoxicity Assay Kit (Beyotime, China). Additionally, after 1, 4, and 7 days of co-culture, the cell viability of chondrocytes was assessed using the Cell Counting Kit-8 (CCK-8) assay.

***Osteoarthritis Cell Model:***

To simulate the chronic inflammatory environment of osteoarthritis (OA), rat chondrocytes were seeded at a density of 2 × 10⁴ cells/mL in the lower chamber of a Transwell and exposed to 10 ng/mL IL-1β (Sigma-Aldrich, USA) for 48h. Different groups of BMSC/MS were injected into the upper chamber for 48h co-culture to assess the therapeutic effect of BMSC^motif+miR874^/MS on damaged chondrocytes in OA. Additionally, chondrocytes that were not treated with IL-1β served as the CTRL group.

***Detection of mRNA Expression in Chondrocytes:***

The chondrocyte samples in the lower chamber of the Transwell were washed three times with PBS, and the remaining liquid was aspirated. Total cellular RNA was then extracted using TRIzol reagent (Invitrogen, USA), followed by reverse transcription using the Prime Script RT reagent Kit (Takara, Japan). Next, a real time PCR machine (Applied Biosystems, ABI7500, USA) and an ABI SyBr Green system were used to perform qPCR. The results were analyzed using "QuantStudio7Flex" software. Gene expression was normalized to β-actin, and relative gene expression was quantified using the ^2-ΔΔ^CT method. Table S2 lists the primers used for qPCR.

**Table S2** Primers used in this experiment.

| Gene and primer direction | Primer sequence (5′to 3′) |
| --- | --- |
| Rat COL2 |  |
| Forward | GACCCCATGCAGTACATG |
| Reverse | GACGGTCTTGCCCCACTT |
| Rat ACAN |  |
| Forward | TGGTGATGATCTGGCACGAG |
| Reverse | CTCCGCTTCTGTAGTCTGCG |
| Rat MMP13 |  |
| Forward | GCTGGAGGATGAGGAGGATGA |
| Reverse | GGAGGAGGAGGAGGAGGAGGA |
| Rat β-actin |  |
| Forward | TGGAATCCTGTGGCATCCATGAAAC |
| Reverse | TAAAACGCAGCTCAGTAACAGTCCG |

***Immunofluorescence Staining of Chondrocytes:***

For the chondrocyte samples as described earlier, immunofluorescence staining was performed using primary antibodies specifically targeting COL2 (abcam, ab185430) and MMP13 (abcam, ab315267), respectively, followed by incubation with goat anti-rabbit IgG (abcam, ab150080) secondary antibody. Finally, the cytoskeleton of the chondrocytes was stained with Actin-Tracker Green-488, and the cell nuclei were stained with DAPI for cellular localization.

***Western Blot:***

For the chondrocyte samples as described earlier, proteins were extracted using RIPA lysis buffer (Sigma, USA). The protein concentration was then determined using the BCA assay. Subsequently, each protein lysate was subjected to sodium dodecyl sulfate-polyacrylamide gel electrophoresis (SDS-PAGE) on a 10% gel, followed by transfer to a polyvinylidene fluoride (PVDF) membrane (Millipore, USA). The membrane was first blocked with 5% skim milk in TBST solution for 1 hour, then incubated overnight at 4°C with primary antibodies against β-actin (CST, 4970), COL2 (CST, 36276), ACAN (CST, 28971), and MMP13 (CST, 67329). After thorough washing, the membrane was incubated with the corresponding secondary antibodies. Chemiluminescent detection was performed using ECL substrate (Thermo Fisher Scientific) to visualize the protein bands.

***Detection of miRNA-874 Expression in Chondrocytes:***

The chondrocyte samples in the lower chamber of the Transwell were washed three times with PBS, and the remaining liquid was aspirated. Total cellular RNA was then extracted using TRIzol reagent, followed by reverse transcription using the Prime Script RT reagent Kit. Next, a real time PCR machine and an ABI SyBr Green system were used to perform qPCR. The results were analyzed using "QuantStudio7Flex" software. Gene expression was normalized to miR-39, and relative gene expression was quantified using the ^2-ΔΔ^CT method. The primers used for qPCR were consistent with Table S1.

***Detection of ROS Expression in Chondrocytes:***

After removing the culture medium from the chondrocytes of each group and washing with PBS, DCFH-DA (Beyotime, China) working solution was added, and the cells were incubated at 37°C with 5% CO₂ for 30 minutes. After incubation, the staining solution was removed, and the cells were washed 2-3 times with PBS to remove any unincorporated DCFH-DA. Next, Hoechst 33342 (Beyotime, China) solution (10 µg/mL) was added, and the cells were incubated at 37°C with 5% CO₂ for 5 minutes. The cells were then washed to remove excess Hoechst solution. Finally, imaging was performed using CLSM, and fluorescence intensity was analyzed using Image J software.

***Evaluation of Mitochondrial Membrane Potential in Chondrocytes:***

Mitochondrial membrane potential was assessed using the JC-1 assay kit (Beyotime, China). The chondrocyte samples, as described earlier, were washed three times with fresh PBS and then incubated with JC-1 solution at 37°C for 30 minutes. Imaging was performed using CLSM, and the fluorescence intensity of JC-1 monomers and aggregates was quantified using ImageJ. The red-to-green fluorescence ratio was further analyzed to evaluate the mitochondrial membrane potential.

***Mitochondrial imaging Analysis:***

After preparing the chondrocyte samples from each group, the cells were gently washed three times with fresh PBS and then incubated with Mito Tracker Red CMXRos (Beyotime, China) for 30 minutes. The cells were subsequently washed three times with fresh PBS and visualized using confocal laser scanning microscopy (CLSM). Quantitative analysis was performed using Image J. For Mito Tracker Red, excitation wavelength (λex) = 579 nm and emission wavelength (λem) = 599 nm.

***Detection of mitochondrial structure in chondrocytes:***

After culturing the chondrocyte samples from each group, the culture medium was discarded, and the cells were washed three times with PBS. The cells were then fixed with 1 mL of 2.5% glutaraldehyde at room temperature for 5 minutes. Using a cell scraper, the cells were collected into a 1.5 mL centrifuge tube and centrifuged at 1000 rpm for 2 minutes. The glutaraldehyde solution was replaced with fresh glutaraldehyde, and the cells were resuspended and fixed again at room temperature for 30 minutes, followed by storage at 4°C. The cell suspension was then dropped onto a 150-mesh copper grid with a formvar film and stained with a 2% uranyl acetate saturated alcohol solution. The copper grid was placed in a grid box and allowed to dry overnight at room temperature. Images were captured using a transmission electron microscope (TEM, HT7800, Hitachi).

***Transcriptome Sequencing Analysis:***

The inflammation-stimulated chondrocytes without treatment and co-cultured with BMSC^motif+miR874^/MS were subjected to transcriptome sequencing and analyzed by OE Biotech Co., Ltd. located in Shanghai, China. Additionally, qPCR was performed to detect the expression of Ikbke between the two groups of chondrocytes. Table S3 lists the primers used for qPCR.

**Table S3** Primers used in this experiment.

| Gene and primer direction | Primer sequence (5′to 3′) |
| --- | --- |
| Rat Ikbke |  |
| Forward | GAACACGTGCCAACAGACAC |
| Reverse | TCCTGCATGTGGAAGACCAG |
| Rat β-actin |  |
| Forward | TGGAATCCTGTGGCATCCATGAAAC |
| Reverse | TAAAACGCAGCTCAGTAACAGTCCG |

***Detection of mRNA Expression in BMSC clusters and BMSCs:***

BMSC clusters and BMSCs were subjected to 14 days of IL-1β-stimulated chondrogenic differentiation induction culture, respectively. mRNA was extracted from BMSC clusters and BMSCs in the Transwell lower chamber using the method described earlier, followed by qPCR analysis. Gene expression was normalized to β-actin, and relative gene expression was quantified using the ^2-ΔΔ^CT method. Table S4 lists the primers used for qPCR.

**Table S4** Primers used in this experiment.

| Gene and primer direction | Primer sequence (5′to 3′) |
| --- | --- |
| Rat SOX9 |  |
| Forward | CACACTACAGCCCCTCCTAC |
| Reverse | CCTCCTCAAGGTCGAGTGAG |
| Rat COL2 |  |
| Forward | GACCCCATGCAGTACATG |
| Reverse | GACGGTCTTGCCCCACTT |
| Rat ACAN |  |
| Forward | TGGTGATGATCTGGCACGAG |
| Reverse | CTCCGCTTCTGTAGTCTGCG |
| Rat β-actin |  |
| Forward | TGGAATCCTGTGGCATCCATGAAAC |
| Reverse | TAAAACGCAGCTCAGTAACAGTCCG |

***Immunofluorescence Staining of BMSC clusters and BMSCs:***

First, BMSC clusters from each group were fixed and blocked, followed by staining with an antibody targeting COL2 (abcam, ab185430). Afterward, the cells were incubated with the corresponding secondary antibody and counterstained with DAPI for 10 minutes. The samples were then imaged in 3D using CLSM.

For the BMSCs in the Transwell lower chamber from each group, the cells were fixed and blocked, then stained with antibodies targeting COL2 (abcam, ab185430) and SOX9 (proteintech, 67439-1-Ig). After incubation with the corresponding secondary antibodies, the cells were counterstained with DAPI for 10 minutes and observed under CLSM.

***Osteoarthritis Rat Model:***

The animal experiments in this study were approved by Jiagan Experimental Animal Management Ethics Committee (Approval No.: JGLL-20231231). Twelve-week-old male Sprague-Dawley (SD) rats were divided into two groups: the sham surgery group (n = 5) and the osteoarthritis (OA) group (n = 15). OA rats underwent medial meniscus resection surgery under anesthesia with 3% sodium pentobarbital (40 mg/kg). Post-surgery, rats were treated with antibiotics (penicillin, 100,000 units intramuscularly daily) and analgesics (carprofen, 1 mg daily, administered as a gel) to prevent post-operative infection and wound licking for 3 days. Four weeks after surgery, OA rats were further randomly divided into three subgroups (n = 5 per subgroup) and received intra-articular injections of 20 µl PBS, BMSC/MS (2.5 mg/mL PBS, 20 µl), or BMSC^motif+miR874^/MS (2.5 mg/mL PBS, 20 µl), respectively, with injections repeated every 2 weeks.

***In vivo live imaging test:***

First, the O-linked glycoproteins on the surface of BMSCs were chemically conjugated with tetraacylated N-azidoacetyl galactosamine (Ac4GalNAz). Then, through the strain-promoted azide-alkyne cycloaddition (SPAAC) reaction, Ac4GalNAz was conjugated with Cyanine7-DBCO, resulting in Cy7-labeled BMSCs. Four weeks after OA modeling, the respective groups of Cy7-labeled BMSCs or BMSC clusters were injected into the joint cavity. In vivo imaging was performed on day 1 and 7 post-injection to observe their local retention capability.

***Radiological evaluation:***

8 weeks after modeling, the rats in each group were anesthetized. X-ray imaging of the right knee joint was performed in the anteroposterior and lateral views, respectively. Based on the X-ray images, the joint space width (JSW) and signs of joint degeneration were evaluated and statistically analyzed.

***Histology and Immunofluorescence Detection:***

After completing the radiological evaluations, the specimens were stored in 4% paraformaldehyde. Following gradient dehydration and decalcification, the samples were embedded in paraffin, and 5 µm sagittal sections were prepared with the repair site at the center. The sections were then stained with H&E, Safranin O/Fast Green, and Toluidine Blue according to the manufacturer’s protocols to assess morphology and GAG distribution. For immunofluorescence staining of COL2 and MMP13, the sections were incubated with anti-COL2 and anti-MMP13 antibodies following the manufacturer’s instructions.

***Detection of miRNA-874 Expression in Articular Cartilage:***

After the tissue section preparation as described earlier, proteinase K digestion was performed to remove DNA and proteins from the tissue, thereby enhancing the hybridization signal. The reaction was terminated with a glycerol solution, and the sections were washed. Following this, a pre-hybridization treatment was carried out by incubating the sections in a pre-hybridization solution containing 5×SSC, 50% phenol/glycerol, and other components in a water bath at 50–60°C for 1 hour. Next, a hybridization solution was prepared by mixing Cy5.5-labeled miRNA-874 probe with the pre-hybridization solution and incubating overnight at 60°C. After hybridization, the sections were washed with 2×SSC for 10 minutes, 0.5×SSC for 15 minutes, and 0.1×SSC for 30 minutes. Finally, the sections were mounted with DAPI-containing mounting solution and observed under a fluorescence microscope to assess the expression of miRNA-874.

***In vivo biocompatibility assessment:***

The main organs (heart, liver, spleen, lung, kidney) from each group of rats were collected and processed into paraffin-embedded sections using the previously described method. The sections were then stained with H&E to evaluate the condition of the main organs under different treatments.

Figure S1: Characterization of GelMA hydrogel microspheres. (a) SEM images of GelMA hydrogel microspheres. (b) Particle size and (c) Pore size analysis of GelMA hydrogel microspheres. (d) The microscopic images demonstrated the morphological change of degradation of GelMA hydrogel microspheres. (e) Degradation rate of GelMA hydrogel microspheres (n = 3). (f) Rheological properties of GelMA hydrogel. (g) Injectability of GelMA hydrogel microspheres.


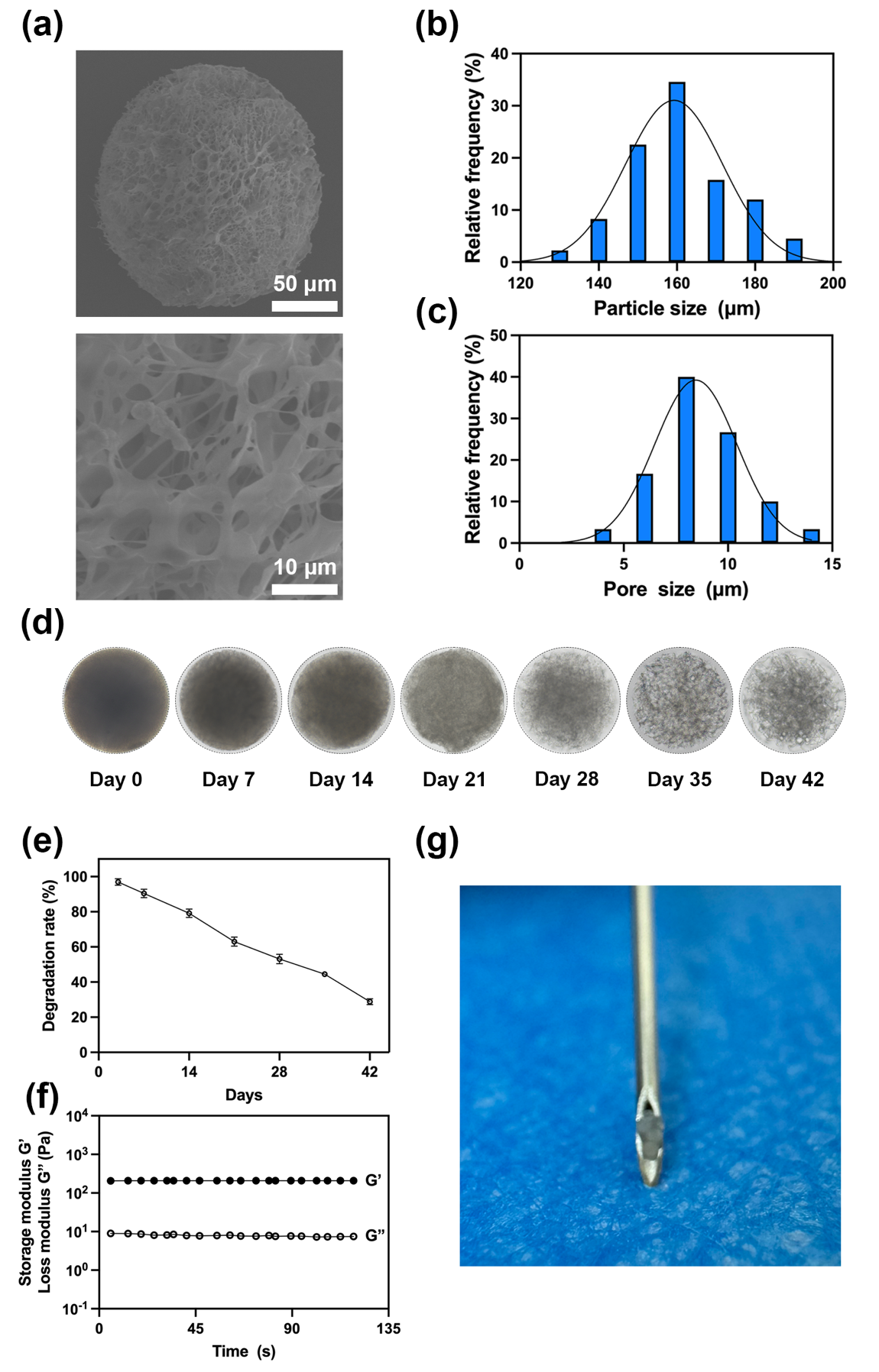


Figure S2: Activity assay of BMSCs and BMSC^motif+miR874^ at various time. Data are presented as mean ± SD (n = 5, ns indicated p > 0.05), with “n” denoting biologically independent experiments. Statistical tests were analyzed by a two-tailed Student's t-test.


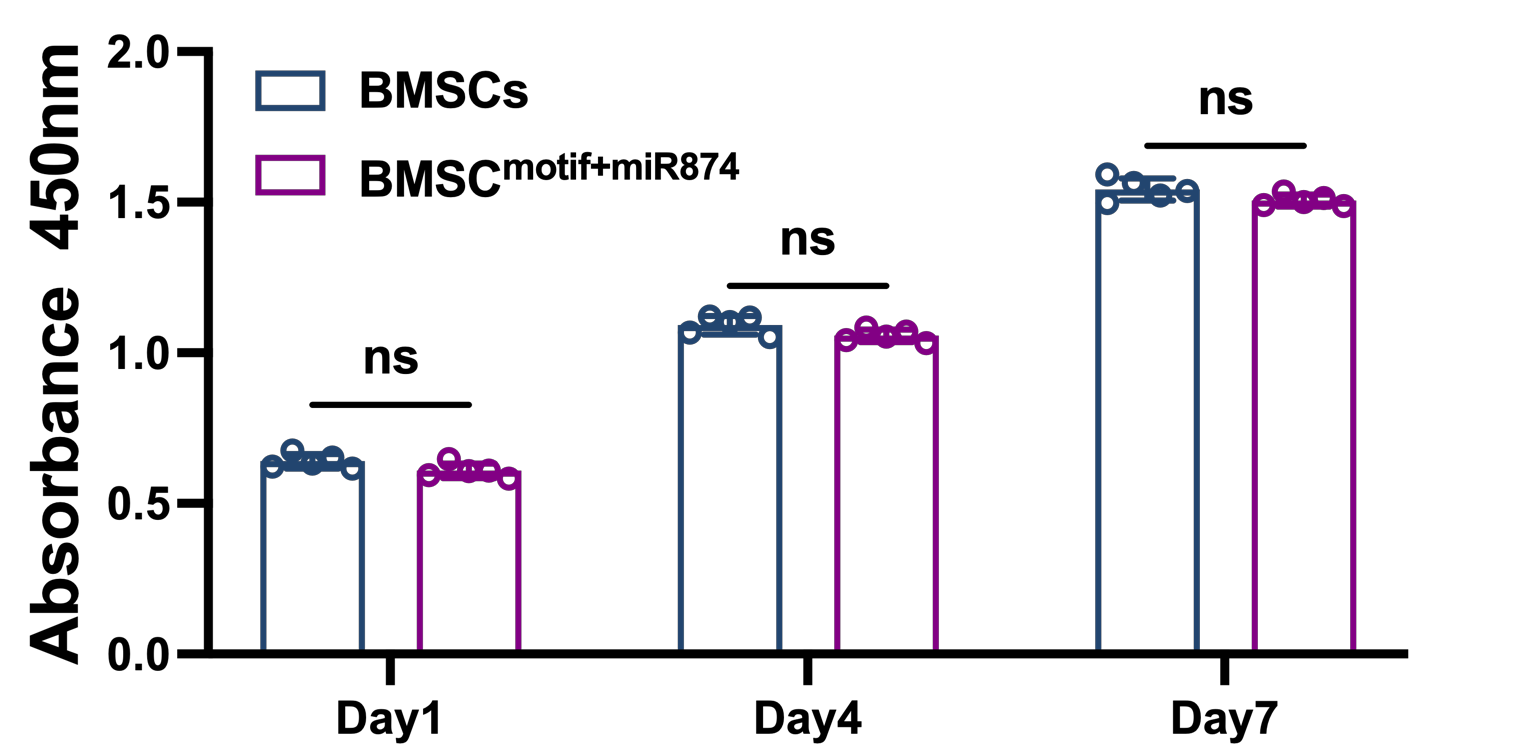


Figure S3: Evaluation of the biocompatibility of BMSC/MS systems *in vitro*. (a) Live-dead staining of chondrocytes co-cultured with different BMSC/MS systems (Scale bar: 100 μm); (b) Activity assay of chondrocytes co-cultured with different BMSC/MS systems at various time Data are presented as mean ± SD (n = 5, ns indicated p > 0.05), with “n” denoting biologically independent experiments. Statistical tests were analyzed by one-way ANOVA with Tukey's post hoc test.


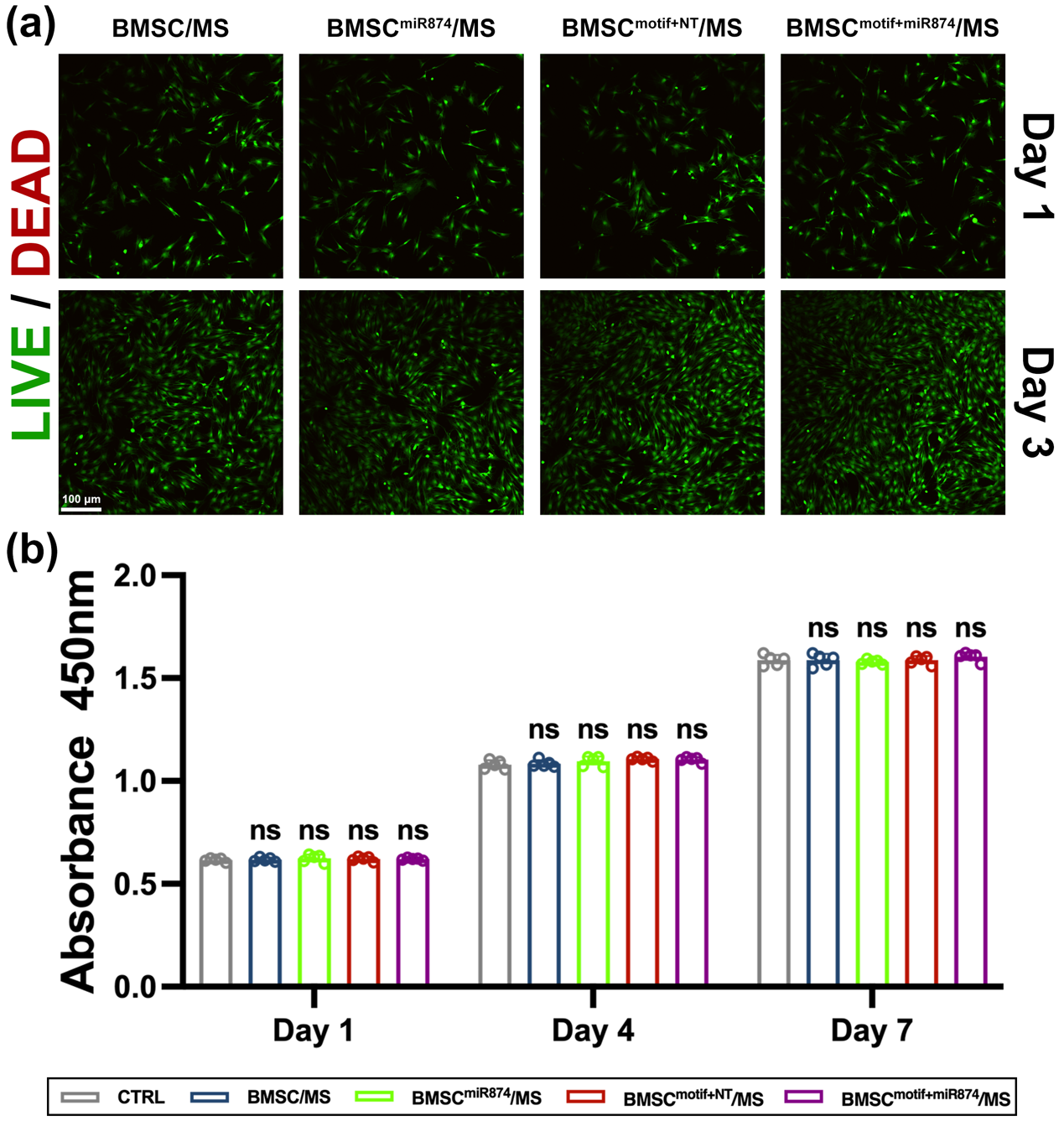


Figure S4: Effects of exosomes alone on protects chondrocyte extracellular matrix. (a) qPCR analysis results of COL2, ACAN, and MMP13. (b) Western blot results of COL2, ACAN, and MMP13 protein. (c) Representative immunofluorescence images of COL2 and MMP13 protein (Scale bar: 50 μm). (d) Relative protein expression analysis of Western blot results. (e) Relative fluorescence intensity quantification of COL2 and MMP13. Data are presented as mean ± SD (n = 5, ns indicated p > 0.05), with “n” denoting biologically independent experiments. Statistical tests were analyzed by a two-tailed Student's t-test.


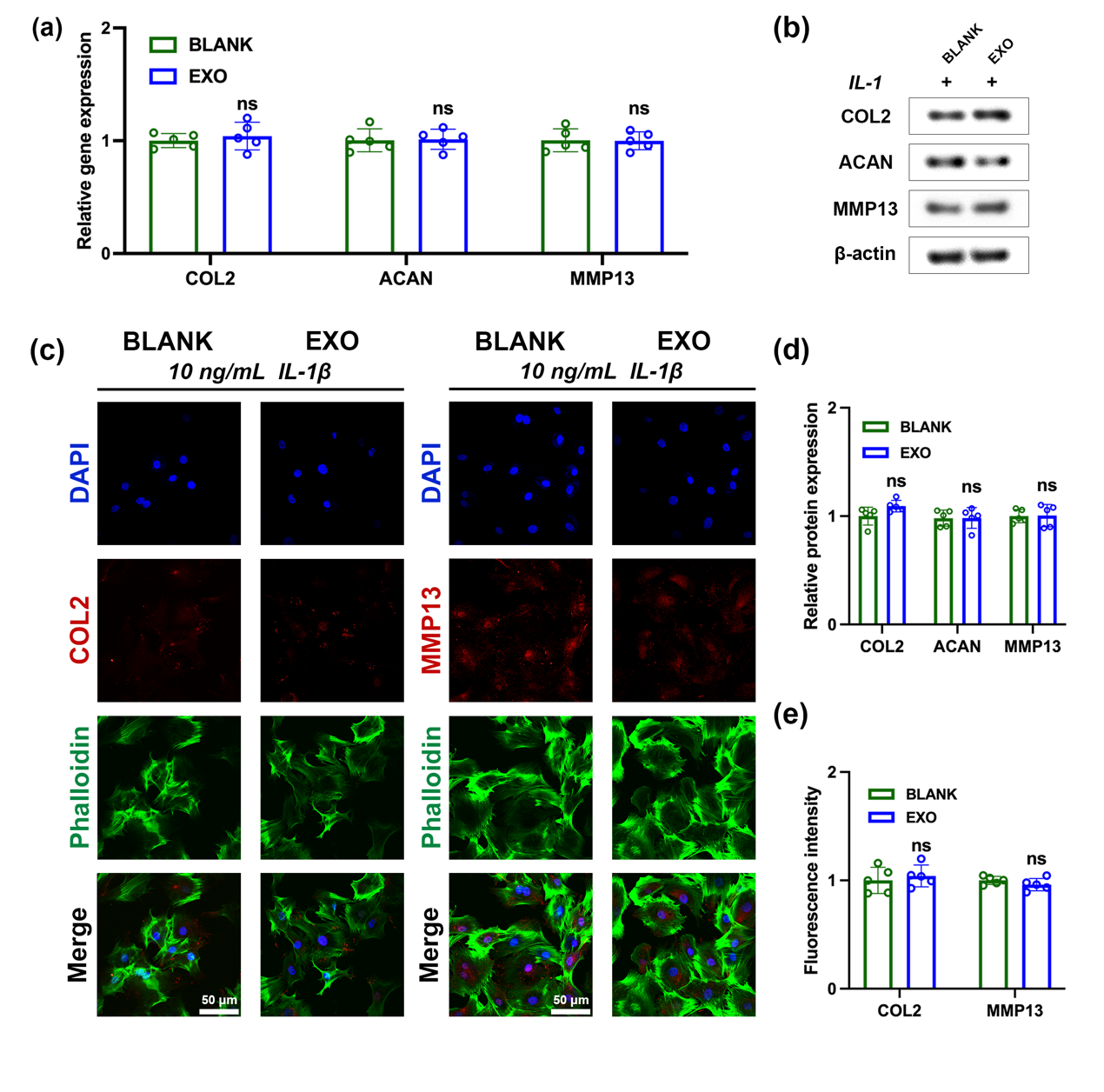


Figure S5: qPCR detection of miR874 expression in chondrocytes of each group. Data are presented as mean ± SD (n = 5, ✱✱✱✱ indicated p < 0.0001), with “n” denoting biologically independent experiments. Statistical tests were analyzed by one-way ANOVA with Tukey's post hoc test.


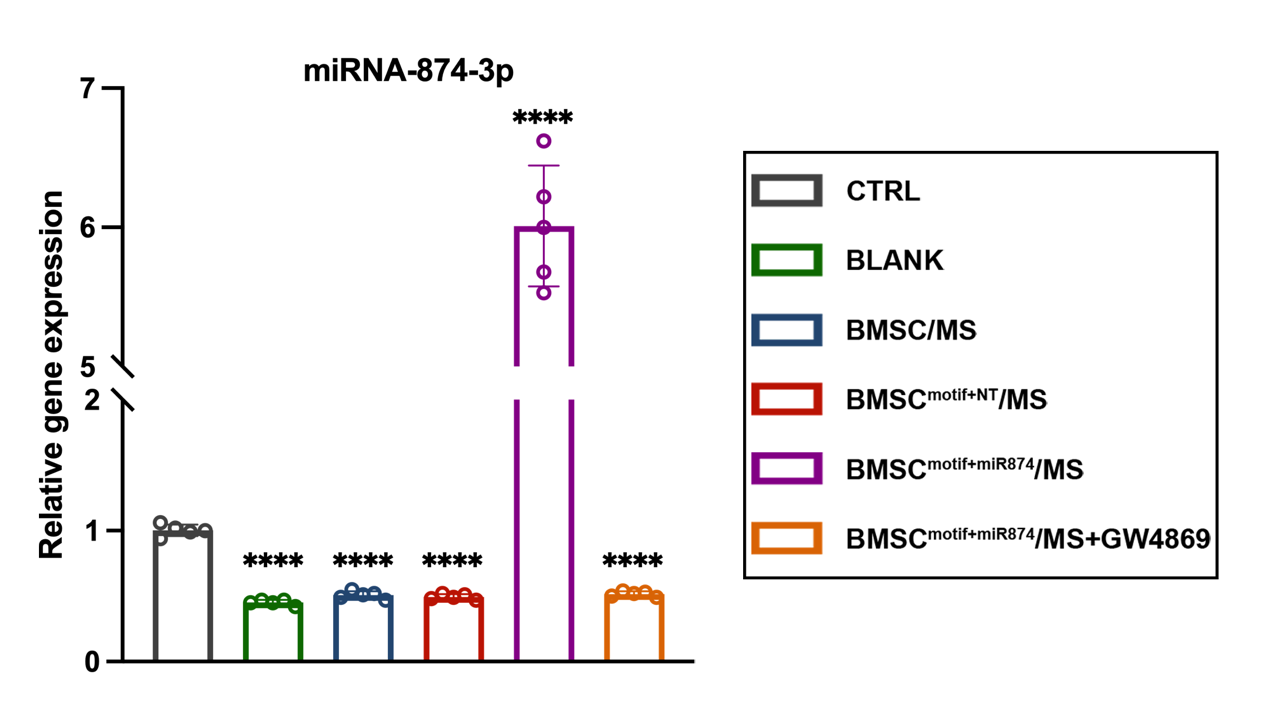


Figure S6: Results of transcriptome sequencing. (a) Correlation analysis of gene expression levels between samples in cells co-cultured with BMSC^motif+miR874^/MS; (b) PCA of the global genes in cells co-cultured with BMSC^motif+miR874^/MS


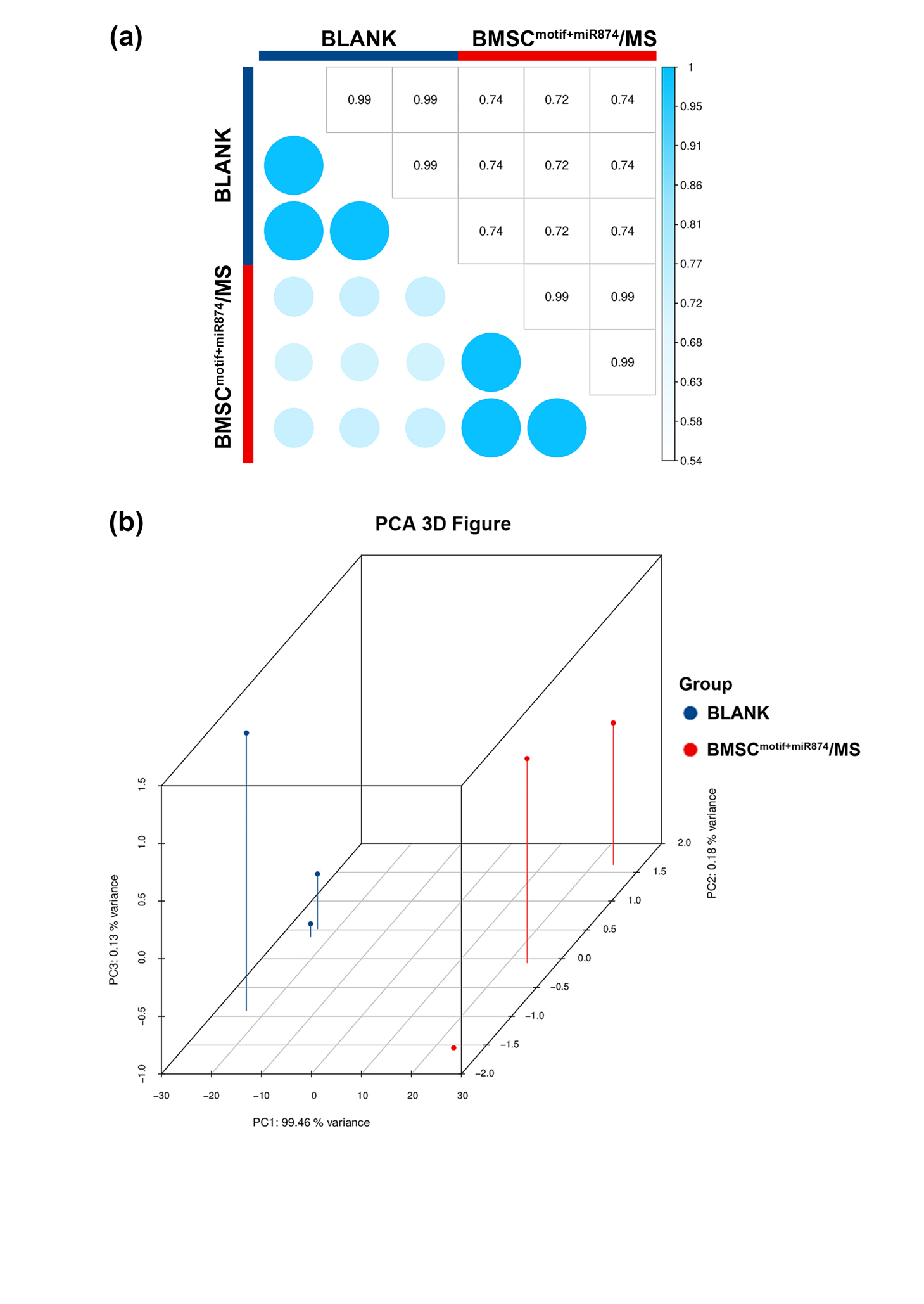


Figure S7: Chord of Top 10 Upregulated classiﬁcations of DEGs According to GO Enrichment Analysis


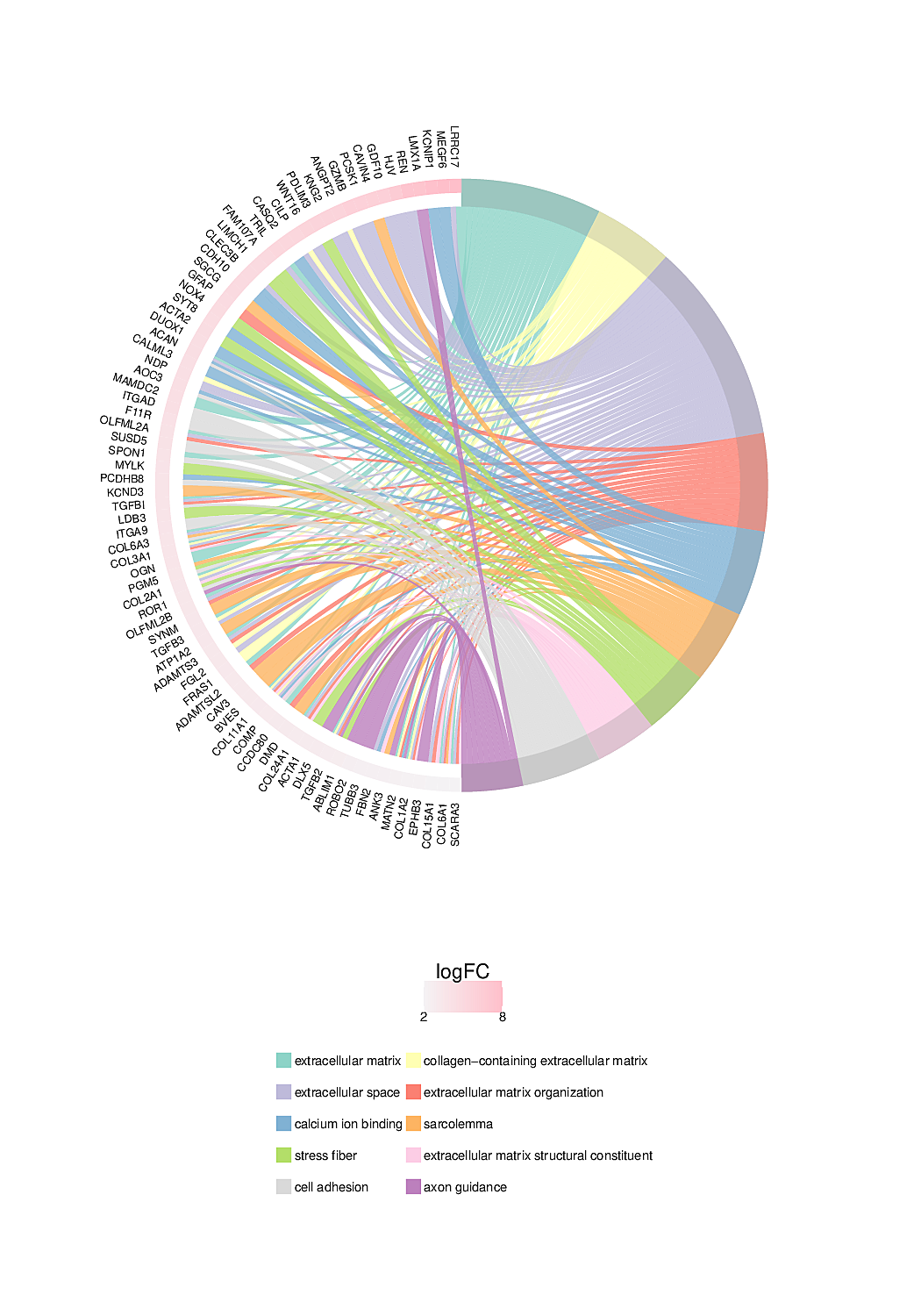


Figure S8: GSEA of Toll-like receptor signaling pathway.


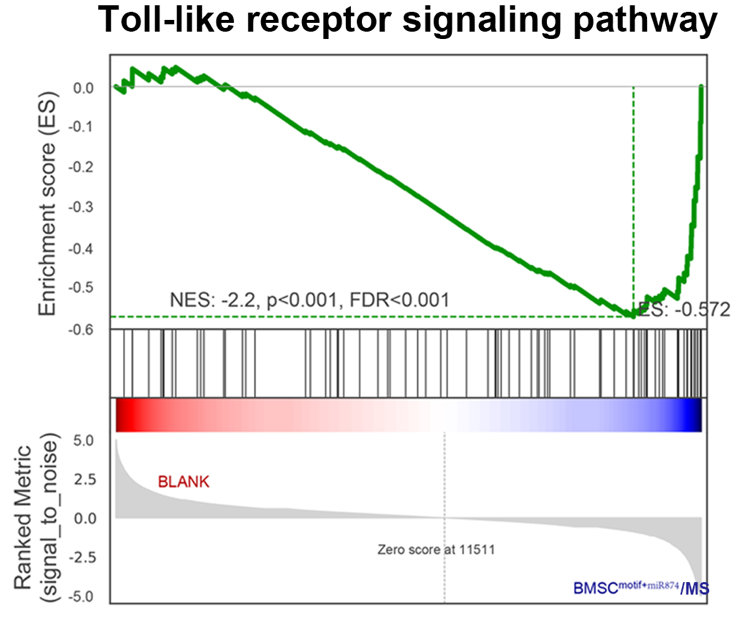


Figure S9: Quantitative analysis of the attenuation rates according to the signals of the radiant efficiency. Data are presented as mean ± SD (n = 5, ✱✱✱✱ indicated p < 0.0001), with “n” denoting biologically independent experiments. Statistical tests were analyzed by one-way ANOVA with Tukey's post hoc test.


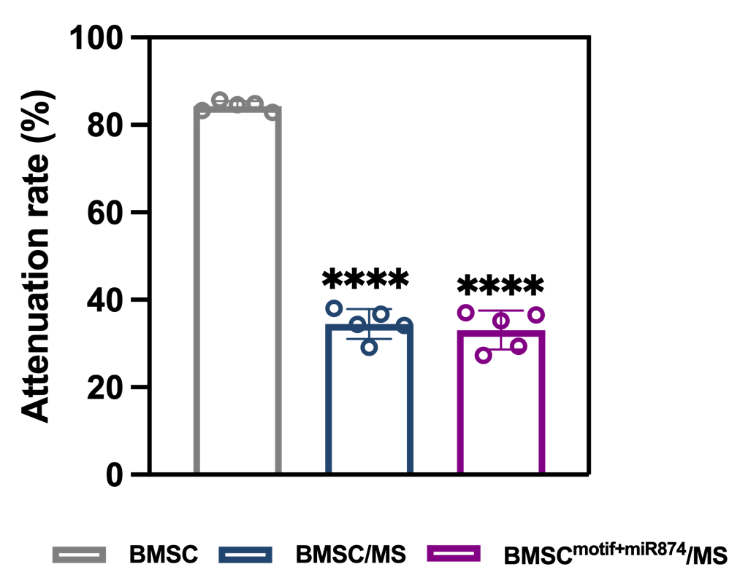


Figure S10: Histological evaluation of the main organs of each group of experimental animals. (Scale bar: 100 μm)


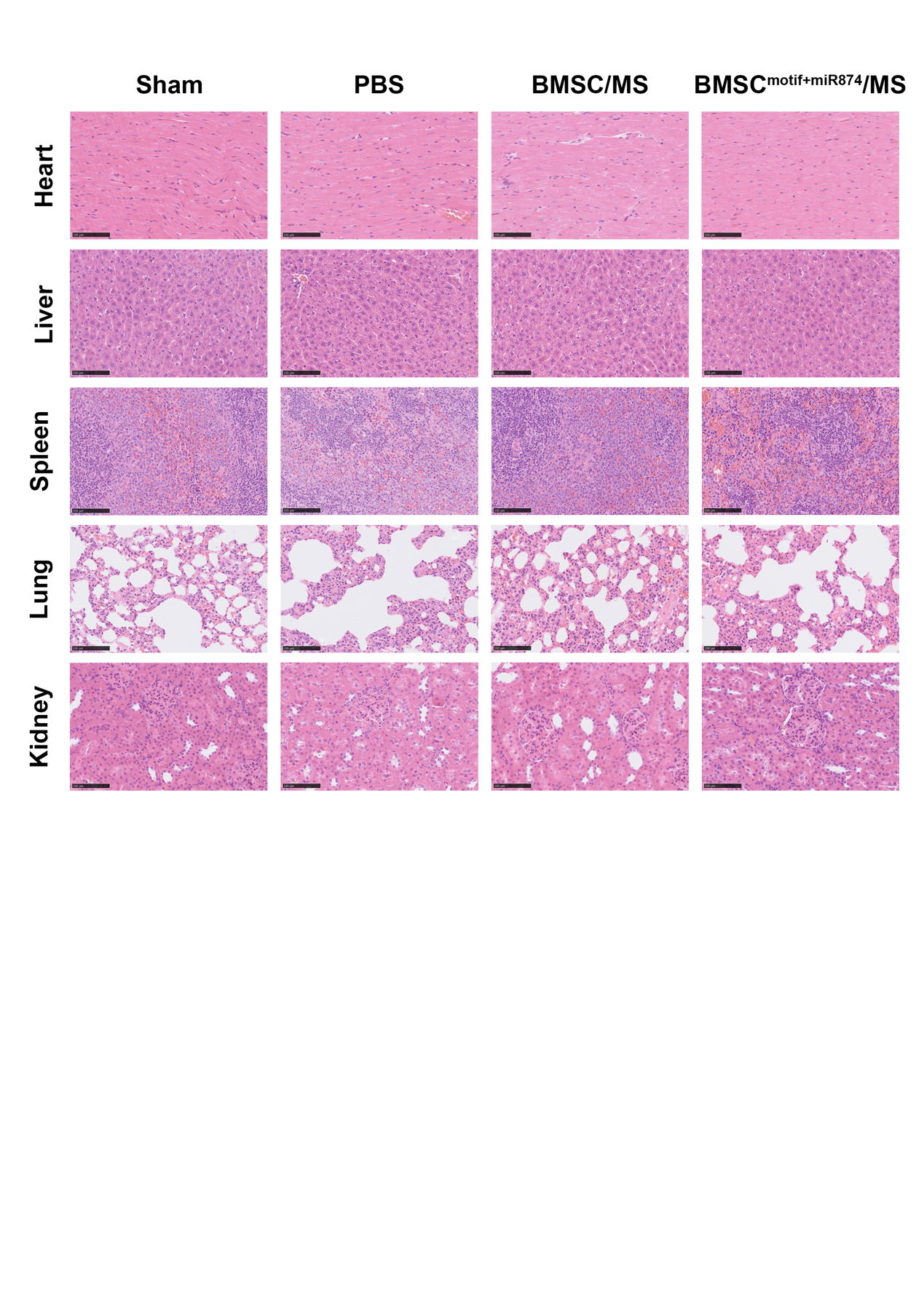

Supplement: Supplementary file 1 — Supporting Information [file ADVS-12-2500964-s001.docx]
